# Impact of climate change on Colombian Pacific coast mangrove bivalves distribution

**DOI:** 10.1016/j.isci.2024.110473

**Published:** 2024-07-10

**Authors:** John Josephraj Selvaraj, Cristiam Victoriano Portilla-Cabrera

**Affiliations:** 1 Universidad Nacional de Colombia, Palmira Campus, Department of Engineering, Faculty of Engineering and Administration, Research Group on Hydrobiological Resources, Carrera 32 No. 12-00 Chapinero, Vía Candelaria, Palmira, Valle del Cauca 763533, Colombia; 2 Universidad Nacional de Colombia, Tumaco Campus, Institute of Pacific Studies, Kilómetro 30-31, Cajapí Vía Nacional Tumaco-Pasto, Tumaco, Nariño 528514, Colombia

**Keywords:** Environmental science, Global change, Zoology, Aquatic biology, Aquaculture

## Abstract

The mangrove bivalves, *Anadara tuberculosa* and *Anadara similis*, are pivotal for the Colombian Pacific coast mangrove ecosystems and economies. In this study, the current and future potential distribution of these bivalves is modeled considering climate change. The future models (2030 and 2050) were projected considering the new climate scenarios (SSP1, SSP2, and SSP5) proposed by the IPCC in its sixth report. Our findings reveal areas in the Colombian Pacific coast, notably Nariño, Cauca, southern Valle del Cauca, and Chocó, with high environmental suitability for these bivalves. However, the 2050 projections, especially under the pessimistic SSP5 scenario, indicate potential adverse impacts from climate change. By 2030 and 2050, the species might lean more toward a southwesterly distribution in the Colombian Pacific coast. Climate-induced spatiotemporal mismatches could occur between the bivalves and the mangroves in some areas. These insights are crucial for effective conservation and management strategies for these species.

## Highlights

• Mangrove distribution predicts Piangua distribution on the Colombian Pacific coast
• Nariño coast has extensive areas suitable for mangrove bivalves’ current distribution
• Climate change affects Piangua distribution, especially under SSP5 scenarios
• Chocó and Nariño will lose extensive areas suitable for female and male Piangua, respectively

## Graphical abstract

**Figure:**
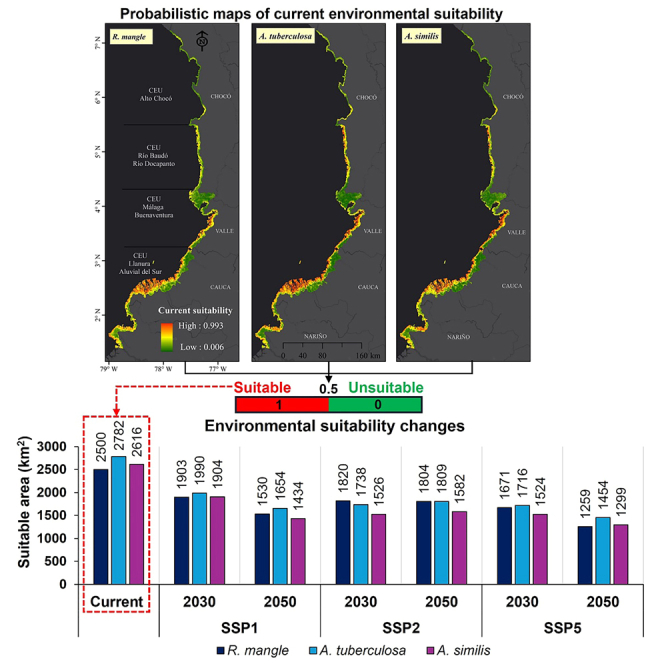

## Introduction

The species *Anadara tuberculosa* and *Anadara similis*, also known as mangrove bivalves, are culturally, commercially, and naturally significant to the Colombian Pacific coast.^1^ Both female (*A. tuberculosa*) and male (*A. similis*) inhabit mud sediments in mangrove forest swamps, primarily beneath the red mangrove *Rhizophora mangle*, across the Pacific from Baja California to Tumbes in Peru.^2^ In Colombia, these bivalves (Spanish common name “Piangua”) span the Pacific coast, from the northern part of the Chocó department (Punta Ardita) to the southern part of the Nariño department (Candelilla de la Mar).^1^

The bivalve extraction, particularly by women, supports about 11,300 Afro-descendant families as a source of food, economy, and cultural livelihood.^3^ These communities face numerous stressors.^4^^,^^5^^,^^6^^,^^7^^,^^8^ Significantly, Nariño and Valle del Cauca boast the highest Catch Per Unit Effort (CPUE), which indicates the average extraction per individual per day, with figures of 160 and 152 bivalves, respectively. These regions also have the highest counts of Piangüeras (locals traditionally associated with hand-collecting bivalve molluscs), numbering 9,040 in Nariño and 1,522 in Valle del Cauca.^3^

Despite significant CPUE values for mangrove bivalves across the Colombian Pacific coast, decreases in their size and quantity and shifts in collection times, seasons, and areas have been reported.^1^ Reductions in bivalve density and distribution areas are driven mainly by changes in habitat extent and quality.^9^ Notably, *A. tuberculosa* is classified as vulnerable and threatened by anthropogenic factors like overfishing and natural factors such as climate change.^1^^,^^10^^,^^11^^,^^12^ There is currently insufficient information to evaluate *A. similis'* threat category in the country.^12^

Scientific evidence underscores that climate change adversely influence oceanic and continental ecosystems, leading to alterations in the intensity, frequency, and duration of extreme events such as winter and/or dry seasons.^13^^,^^14^ Further research indicates that shift in the precipitation regime and temperature increase are detrimentally impacting mangrove forests.^15^ These changes subsequently affect the composition, distribution, and abundance of bivalves such as the Piangua.^10^

As climate change impacts natural and social systems, the Intergovernmental Panel on Climate Change (IPCC) has projected global temperature increases based on various future climate scenarios. In the sixth report, the IPCC estimated that, under an optimistic scenario (Shared Socioeconomic Pathways [SSP1]) global warming by 2100 could range from 3°C to 3.5°C.^16^ Under intermediate (SSP2) and pessimistic (SSP5) scenarios, it will range from 3.5°C to 3.8°C and 4.7°C to 5.1°C, respectively.^16^ Given the undeniable impact of climate change on natural systems in complex ways,^17^ there is an urgent need to assess its effects on fishery resources such as mangrove bivalves.

On the Colombian Pacific coast, several efforts have been made to study the biological and fishing resources of the Piangua.^10^^,^^18^^,^^19^^,^^20^ However, there is still a need to enhance our understanding of the potential distribution of these bivalves in the face of changing environmental conditions. A series of studies by Agudelo^1^ attempted to model the density distribution of the Piangua along the Colombian Pacific coast, using point interpolation models based on occurrence records of some species collected in various regions of the study area. Nevertheless, these models must be refined by correlating species occurrence data with multiple environmental variables, including soil physicochemical properties, climate change, and the potential future distribution of mangroves. It is important to recognize that climate change poses a latent threat to mangrove ecosystems.^15^ Additionally, the habitat range of species such as *R. mangle*, which provides critical habitat for Piangua, is expected to decline due to future climate changes.^21^

**Figure 1:**
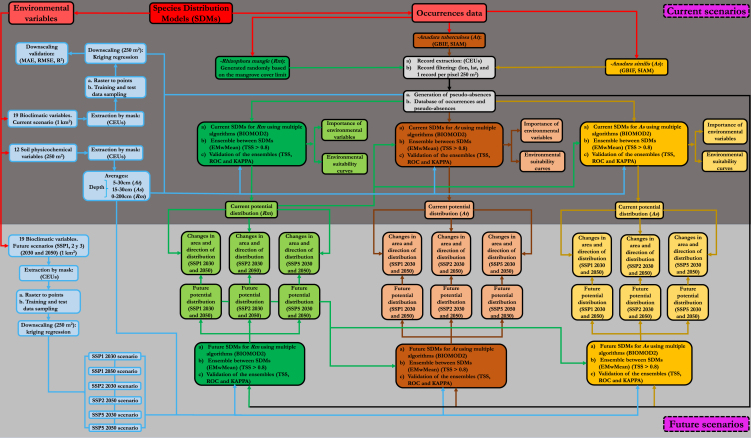
Methodological process for modeling the current and future environmental suitability of Piangua in the Coastal Environmental Units of the Colombian Pacific

Understanding Piangua’s spatial distribution is crucial for its conservation and sustainable use. Hence, there is a need for studies that contribute to the identifying suitable areas for Piangua’s potential distribution in response to environmental changes. In this context, tools such as Species Distribution Models (SDMs) “environmental suitability models” have been suggested (Figure 1). These tools allow predicting the potential distribution of species in geographic space and time, based on correlating the known occurrence data of species with their environmental preferences.^22^^,^^23^ The primary outputs of SDMs are environmental suitability maps of the species, demonstrating the species' probability of occurrence as a function of the environmental features of the geographic area.^24^ The knowledge generated through these tools enables the identification of protected and conservation areas, the assessment of climate change effects, and other analyses.^25^

Different SDMs exist, but a reasonable selection of these is crucial to achieving accurate predictions. A subset of models, including BIOCLIM and Surface Range Envelope (Profile Models [PM]), estimate the environmental suitability of species by taking characterization of the ecological conditions of the areas associated with species occurrences.^26^ Conversely, models like Voronoi Hull, Convex Hull, Inverse-Distance Weighted, and Circles Geographic Distance (Geographic Models [GM]) estimate species presence probabilities based on known occurrence records but do not account for the values of environmental conditions.^26^ Both PM and GM lack a robust integration of the factors involved in species distributions, as these models are predicated on finding one or more decision rules.^27^

**Figure 2:**
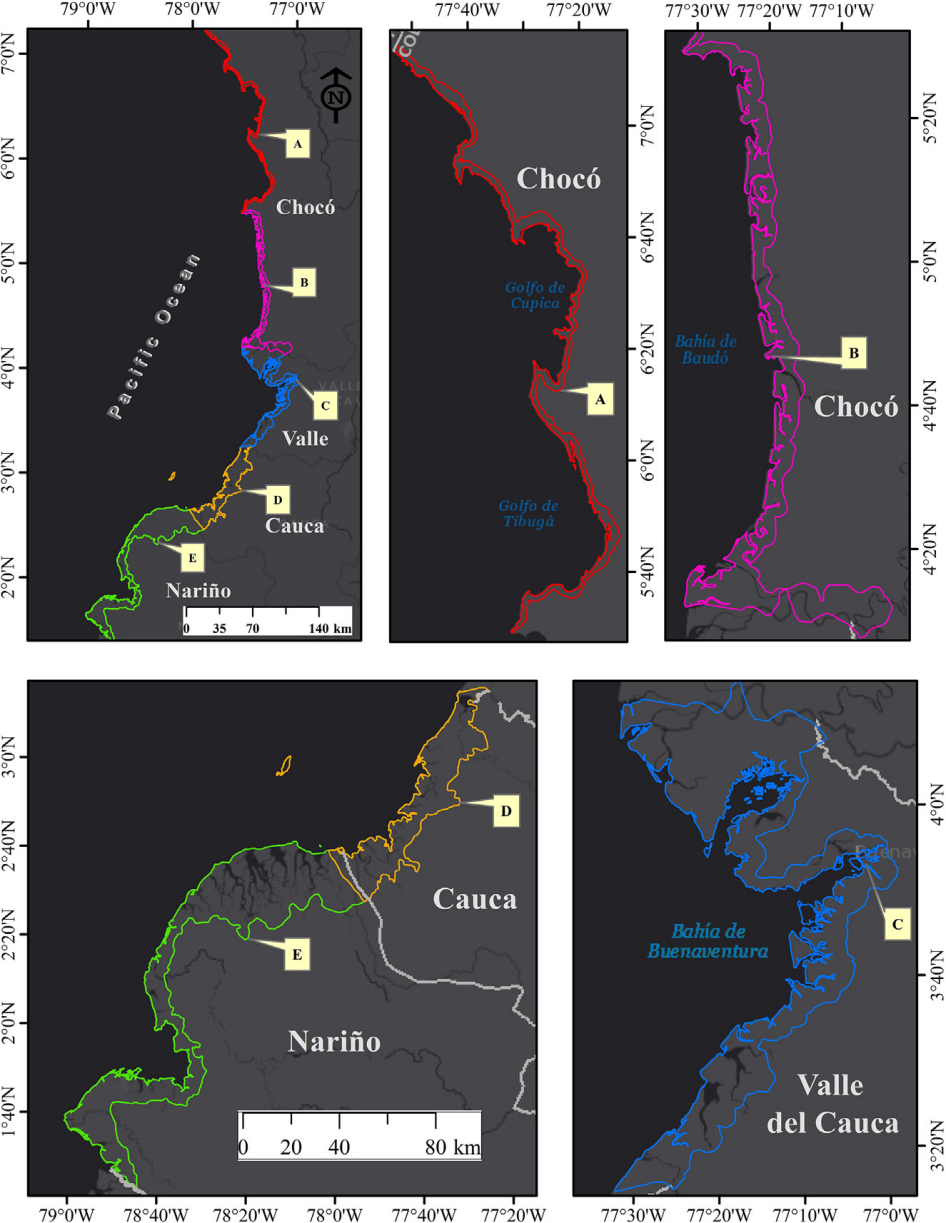
Location of the Coastal Environmental Units associated with the departments of Nariño, Cauca, Valle del Cauca, and Chocó in the Colombian Pacific Coastal areas of northern Chocó (A), southern Chocó (B), Valle del Cauca (C), Cauca (D), and Nariño (E).

As an alternative to the models described earlier, SDMs based on machine learning have been proposed. Machine learning techniques have been employed to study the distribution of numerous species,^28^^,^^29^^,^^30^ including several marine bivalves. These models include new parameters that can better differentiate environmental appropriateness for species, resulting in higher performance and more accurate predictions.^22^ It was impossible to find research that assess the impact of climate change on the joint distribution of Piangua and mangrove in the Colombian Pacific coast. In this regard, techniques based primarily on machine learning were employed to forecast the possible distribution of the Piangua (*A. tuberculosa* and *A. similis*) and potential habitat (*R. mangle*) under current and future climatic conditions in the Colombian Pacific coast (Figure 2).

## Results and discussion

### Occurrence and pseudo-absence data used

A total of 94, 130, and 100 presence-only data were used to model the potential distribution of *A. similis*, *A. tuberculosa*, and *R. mangle*, respectively. The number of pseudo-absences for each species was equal to the number of occurrences, respectively. The maps related to the location of the occurrence and pseudo-absence data, as well as the preliminary areas of environmental suitability (according to the SRE model) for the Piangua and mangrove (*R. mangle*) are shown in the supplemental information (Figure S1).

### Validation of interpolation models

The interpolation models (kriging regression) used to downscale the projected bioclimatic variables’ spatial scale for the study area’s current and future time periods were accurate (Table S1). When the predictions of the interpolated bioclimatic variables (spatial resolution of 250 m^2^) were validated (2,713 points) against the values of the raw variables (1 km^2^), low values were obtained in the Mean Absolute Error (MAE) and Root-Mean-Square Error (RMSE) tests. Additionally, high values were observed in the test associated with the Coefficient of Determination (R-squared). These results indicate that the predicted values are very close to the actual values.^31^

### Evaluation of the ensembles between SDMs

**Figure 3:**
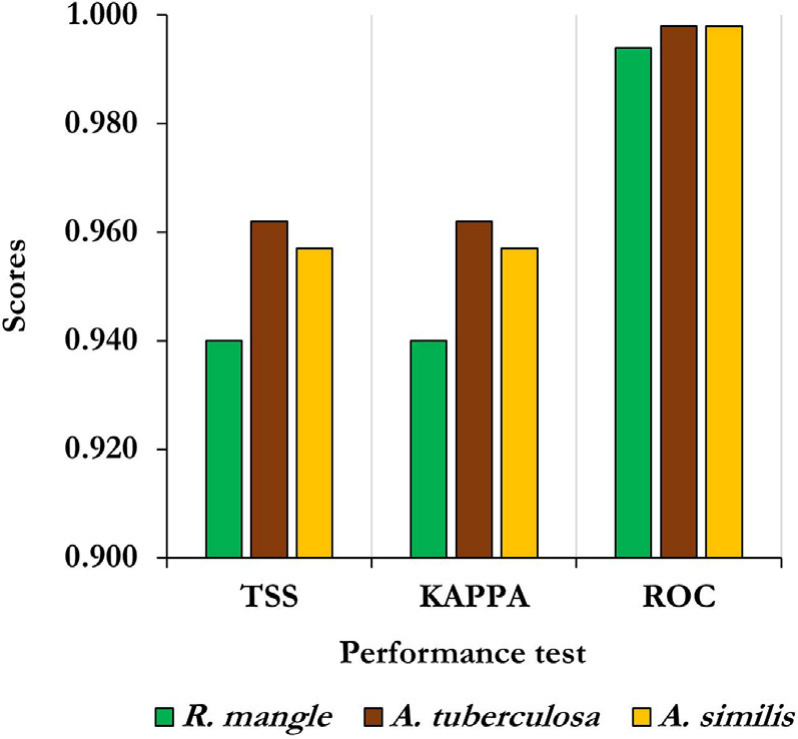
Weighted average performance of the ensemble between the Random Forest (RF) runs to estimate the potential distribution of the Piangua species (*A. tuberculosa* and *A. similis*) and their potential habitat (*R. mangle*) in the Coastal Environmental Units of the Colombian Pacific

Single algorithm models presented different predictive performances. However, the RF models' overall performance was better than the others (Figure S2). Figure 3 shows the evaluation metrics associated with the final ensemble in the implementations of the RF algorithm, which presented a performance greater than 0.9 according to the KAPPA, TSS, and AUC-ROC statistical tests. Maravillas^32^ also showed that RF predicts well the potential habitat of other bivalves.

This result agrees with what Huang^33^ found. They used ensembles made with Biomod2 to make predictions that were very accurate (AUC-ROC, KAPPA, and TSS values greater than 0.90), which made it possible to tell which environments will be best for some plants as the climate changes in the future. Grenouillet^34^ also reported that ensembles between SDMs significantly enhance their performance metrics. Furthermore, these authors validated the hypothesis that distribution models built for specialized species yield higher predictive performance than those applied to generalist species. These findings may be comparable to those in this study, considering that the species of Piangua and mangrove are distributed under specific and restricted environmental conditions.^1^

The findings of this study are consistent with findings of Braunisch,^35^ Harris,^36^ and Porfirio,^37^ who achieved good predictive performance in modeling the future distribution of species of interest for conservation using machine learning models and data from species occurrences along with multiple bioclimatic variables. Liang^38^ further reported that models built with RF have good performance and transferability—ability of the models to predict the potential distribution of species under new and changing conditions.^23^ Consequently, the models developed in this study serve as valuable tools for various purposes, including the planning of species management and conservation efforts in the face of future climate change.

### Importance and response curves of environmental variables

Table 1 shows the relative contribution of environmental variables (Table 2) in the current environmental suitability models for Piangua and mangrove. Furthermore, Table 1 shows the current ranges of environmental suitability where species are more likely to be distributed (≥50%). The environmental suitability ranges were extracted from the response curves associated with bioclimatic and physicochemical suitability of the soil for potential distribution of Piangua and mangrove in Colombian Pacific CEUs (Figures S3–S5).

**Table 1:** Relative contribution of environmental variables in the environmental suitability models for Piangua (*A. tuberculosa* and *A. similis*) and mangrove (*R. mangle*) in the Coastal Environmental Units of the Colombian Pacific

	Contribution to environmental suitability models (%)			Environmental suitability ranges for a potential distribution ≥50%		
Environmental variables [Unit]	(*Rm*)	(*At*)	(*As*)	(*Rm*)	(*At*)	(*As*)
ESRm [%]	∗	38.908	36.135	∗	32–94	39–93
BDOD [cg/cm^3^]	73.155	28.365	33.556	55–106	55–119	54–83
DWB [degrees]	5.418	4.009	5.742	0.002–0.041	0.002–0.034	0.002–0.016
Silt [g/kg]	5.418	3.173	5.893	168–380	269–401	281–375
Nitrogen [cg/kg]	3.983	1.383	0.980	229–482	263–725	201–633
Bio17 [mm]	1.038	2.487	2.599	80–1,265	83–1,299	95–976
Bio14 [mm]	2.344	2.485	1.162	18–396	17–396	20–301
Bio8 [°C]	0.953	2.933	1.221	25–26	25–26	25–26
SOC [dg/kg]	0.953	0.480	2.713	342–1,987	381–1,824	304–1,617
Sand [g/kg]	0.000	1.438	0.951	224–589	216–548	221–632
Bio18 [mm]	0.480	0.858	1.641	170–1,564	170–1,557	189–1,190
Bio9 [°C]	0.427	1.721	0.823	25–27	25–27	25–27
DEM [m]	1.593	0.460	0.780	3–1,064	0–473	0–473
Bio4 [CV]	1.101	0.838	0.726	21–41	29–30	22–40
Ph [pHx10]	0.433	1.782	0.315	48–53	47–57	47–56
Bio10 [°C]	0.378	1.684	0.414	26–27	25–27	26–27
Bio11 [°C]	0.156	1.660	0.489	26–27	24–26	25–26
CFVO [cm^3^/dm^3^](vol %)	0.888	0.313	0.827	27–177	26–158	26–167
Bio1 [°C]	0.187	1.489	0.325	25–27	25–27	25–27
CEC [mmol/kg]	0.420	0.609	0.284	132–355	132–395	125–392
Bio19 [mm]	0.827	0.205	0.225	310–2,286	337–2,326	322–2,265
Bio7 [°C]	1.009	0.111	0.111	7–10	7–10	7–10
Bio6 [°C]	0.263	0.539	0.388	21–23	30–32	21–23
Bio13 [mm]	0.678	0.346	0.139	298–809	229–814	308–816
Bio15 [CV]	0.448	0.197	0.291	19–64	18–64	18–60
Bio16 [mm]	0.618	0.147	0.167	843–2,334	847–2,337	887–2,344
OCD [g/dm^3^]	0.375	0.330	0.162	190–365	264–475	192–316
Bio3 [%]	0.158	0.277	0.430	81–94	83–94	83–94
Bio2 [°C]	0.338	0.155	0.102	7–9	7–9	7–9
Bio12 [mm]	0.214	0.240	0.123	2,323–7,103	2,406–7,055	2,417–7,191
Clay [g/kg]	0.160	0.116	0.221	206–458	215–445	195–451
Bio5 [°C]	0.326	0.094	0.066	30–32	22–40	30–32

The variable ESRm (∗) was included as a predictor of the environmental suitability of the two species of Piangua.

**Table 2:** Environmental variables incorporated into the models to predict environmental suitability for Piangua (*A. tuberculosa* and *A. similis*) and mangrove (*R. mangle*) in the Coastal Environmental Units of the Colombian Pacific

Code	Environmental variables	Unit
Bio1	Annual mean temperature	[°C]
Bio2	Mean diurnal range (mean of monthly [max temp−min temp])	[°C]
Bio3	Isothermality (BIO2/BIO7) (×100)	[%]
Bio4	Temperature seasonality (standard deviation ×100)	[CV]
Bio5	Max temperature of warmest month	[°C]
Bio6	Min temperature of coldest month	[°C]
Bio7	Temperature annual range (BIO5-BIO6)	[°C]
Bio8	Mean temperature of wettest quarter	[°C]
Bio9	Mean temperature of driest quarter	[°C]
Bio10	Mean temperature of warmest quarter	[°C]
Bio11	Mean temperature of coldest quarter	[°C]
Bio12	Annual precipitation	[mm]
Bio13	Precipitation of wettest month	[mm]
Bio14	Precipitation of driest month	[mm]
Bio15	Precipitation seasonality	[CV]
Bio16	Precipitation of wettest quarter	[mm]
Bio17	Precipitation of driest quarter	[mm]
Bio18	Precipitation of warmest quarter	[mm]
Bio19	Precipitation of coldest quarter	[mm]
BDOD (∗)	Bulk density of the fine earth fraction	[cg/cm^3^]
CEC (∗)	Cation exchange capacity of the soil	[mmol/kg]
CFVO (∗)	Volumetric fraction of coarse fragments (>2 mm)	[cm^3^/dm^3^] (vol %)
Clay (∗)	Proportion of clay particles (<0.002 mm) in the fine earth fraction	[g/kg]
Nitrogen (∗)	Total nitrogen (N)	[cg/kg]
OCD (∗)	Organic carbon density	[g/dm^3^]
pH (∗)	Soil pH	[pHx10]
Sand (∗)	Proportion of sand particles (>0.05 mm) in the fine earth fraction	[g/kg]
Silt (∗)	Proportion of silt particles (≥0.002 mm and ≤0.05 mm) in the fine earth fraction	[g/kg]
SOC (∗)	Soil organic carbon content in the fine earth fraction	[dg/kg]
DWB	Distances to water bodies	[degrees]
DEM	Digital elevation model	[m]
ESRm	Environmental suitability for *R. mangle*	[%]

The soil physicochemical variables (∗) were averaged based on the soil depth deemed appropriate for each species. Additionally, the model for Piangua includes a variable related to the environmental suitability for *R. mangle* (ESRm), indicating its integrated approach. The acronym [CV] denotes the coefficient of variation, providing a measure of variability relative to the mean of the variable.

The results suggest that the modeled species share similarities regarding the environmental variables that effectively discriminate their environmental suitability in the study area. The environmental suitability of the mangrove *R. mangle* (ESRm) emerged as a significant variable contributing to the prediction of environmental suitability for Piangua, accounting for 38.9% and 36.1% of the models for *A. tuberculosa* (*At*) and *A. similis* (*As*), respectively. These findings underscore the importance of the mangrove ecosystem (*R. mangle*) in determining the potential distribution of Piangua, reflecting the interdependency observed between these species in their natural habitat.^2^^,^^10^

Bulk density of the fine earth fraction (BDOD) was the second variable with the greatest contribution in predicting the environmental suitability of the Piangua, as its importance in the models built for *At* and *As* was 28.4% and 33.5%, respectively. Other notable physicochemical variables in the discrimination of the distribution of Piangua were distance to water bodies (DWB) and proportion of silt particles in the fine earth fraction (Silt). The importance of the variables DWB (4.1% [*At*] and 5.7% [*As*]), Silt (3.2% [*At*] and 5.9% [*As*]), and BDOD in the suitability models for Piangua is possibly due to the following reasons: Areas with less compacted soils (silty-clay mangrove soils) tend to be more suitable for the potential distribution of Piangua, as it has been identified that mangrove soils with lower bulk density typically harbor higher organic matter and carbon content,^39^ important factors for the development of Piangua.^40^ The significance of the DWB variable is possibly due to it acting as a proxy indicator for salinity and/or flooding, conditions necessary for the natural distribution of Piangua and mangrove.^41^^,^^42^^,^^43^

The influence of bioclimatic variables related to precipitation of the driest quarter (2.5% [*At*] and 2.6% [*As*]) and precipitation of the driest month (2.5% [*At*] and 1.2% [*As*]) on the environmental suitability models for Piangua may be due to the following: When the Colombian Pacific coast undergoes dry seasons, rainfall becomes scarcer, hence salinity tends to increase in mangrove zones.^44^ These results align with those reported by Prado-Carpio,^42^ who noted through a literature review, that the development of Piangua is favored when soils have high concentrations of salinity. Additionally, the contribution of the bioclimatic variable associated with the mean temperature of wettest quarter (2.9% [*At*] and 1.2% [*As*]) could be due to the following: During rainy seasons in the Colombian Pacific, air temperature generally drops.^45^ This is a limiting condition for the potential distribution of the mangrove forest and likely also for species such as Piangua.^42^^,^^46^

### Current environmental suitability

**Figure 4:**
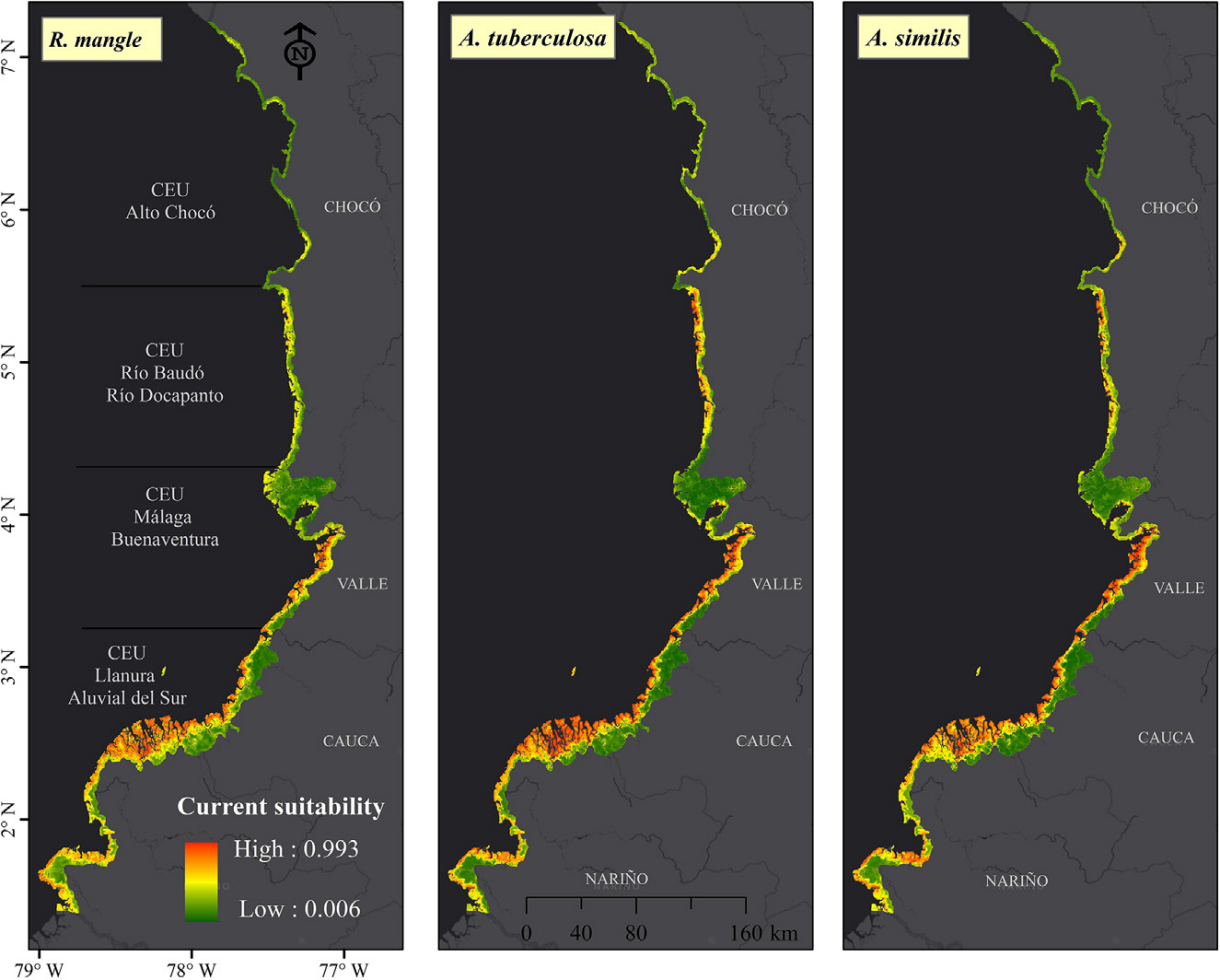
Probabilistic maps of current environmental suitability for Piangua (*A. tuberculosa* and *A. similis*) and mangrove (*R. mangle*) in the Coastal Environmental Units of the Colombian Pacific Areas leaning toward red indicate high environmental suitability for species' distribution, whereas areas leaning toward green suggest low environmental suitability.

Figure 4 shows the current potential distribution of Piangua (*A. tuberculosa* and *A. similis*) and mangrove (*R. mangle*) in the study area. In the current scenario, a large portion of the CEUs associated with the departments of Nariño and Cauca, along with the southern parts of Valle del Cauca and Chocó, display high environmental suitability for the possible distribution of Piangua and mangrove. The suitable area for the potential distribution of the species tended to be greater in the south of the Colombian Pacific coast because in this region there are large areas with environmental conditions potentially more suitable for the potential distribution of the Piangua and the mangrove.^1^ The areas identified in this study are determined by the intersection of suitable abiotic factors for the species and the geographical areas they can ecologically access, free from barriers to movement and colonization.^47^ A species' occurrence depends on factors like historical or dispersal limitations, meaning that a species may not always be found in all potential areas.^48^ While the principles of conservatism and equilibrium between species and their environment are crucial for understanding SDMs,^49^ these principles are subject to the impacts of natural and anthropogenic disturbances affecting mangrove ecosystems in the Colombian Pacific coast.^50^^,^^51^^,^^52^

These results align with the finding of Agudelo,^1^ who used geostatistics and field sampling (161 points) to determine that the average density of *A. tuberculosa* in coastal areas associated with the departments of Nariño, Cauca, Valle del Cauca, and Chocó is approximately 0.93, 0.61, 0.54, and 0.54 individuals/m^2^, respectively. The same authors reported that the density of *A. tuberculosa* is higher than that of *A. similis*. Furthermore, the Piangua density tends to be higher in the southern Colombian Pacific coast, especially in Candelillas de la Mar (1.81 individuals/m^2^), and the north of the department of Nariño (Sanquianga National Park [0.97 ind/m^2^]). Borda and Cruz,^10^ reported that Tumaco’s coastal zones contribute more than 60% of the Piangua production that leaves the country. Meanwhile, the coastal zones associated with the southern parts of Valle del Cauca and Chocó departments (Virudó: 1.50 individuals/m^2^) harbor significant Piangua densities, whereas the northern zone of the Chocó department has lower densities.^1^ The densities of Piangua in the Colombian Pacific coast can vary due to several factors, including fishing pressure on the resource.^3^

According to current projections (Figure 2), mangrove *R. mangle* and Piangua (especially with *A. tuberculosa*) share similar suitable zones in the study area. Mangrove forests play a critical role in establishing food chains, facilitating the flow of matter (carbon) and energy.^53^ Additionally, these ecosystems accumulate organic waste and release nutrients through the decomposition of organic matter, which is crucial for Piangua as it filters organic matter for food.^1^ Simultaneously, mangroves bivalves contribute to the growth and survival of plants by stabilizing and fertilizing sediments and reducing water turbidity.^54^ These findings align with Franco-Vidal,^55^ who observed that the distribution of Piangua populations on the Pacific coast of Chocó is enhanced by the presence of conserved mangrove forests. Moreover, species of *A. tuberculosa* are commonly found among the roots of mangrove forests, especially *R. mangle*, whereas *A. similis* is typically found in more open areas somewhat removed from this ecosystem.^2^ This discrepancy could explain why *A. tuberculosa* exhibits a relatively greater range of environmental suitability than *A. similis*.

### Future environmental suitability changes

**Figure 5:**
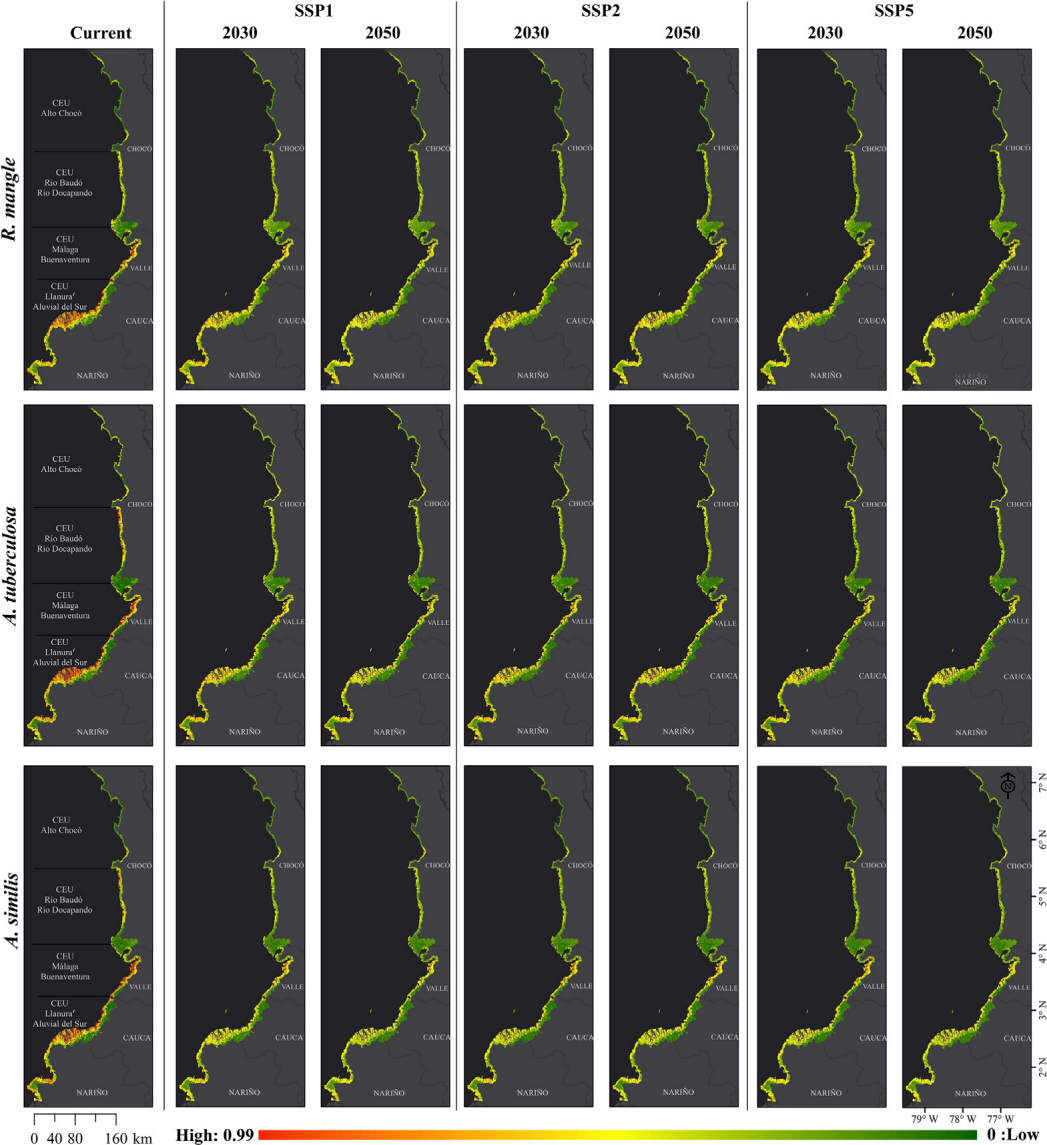
Current and future environmental suitability (2030 and 2050, under climate scenarios SSP1, SSP2, and SSP5) for Piangua (*A. tuberculosa* and *A. similis*) and mangrove (*R. mangle*) in the Coastal Environmental Units of the Colombian Pacific

**Figure 6:**
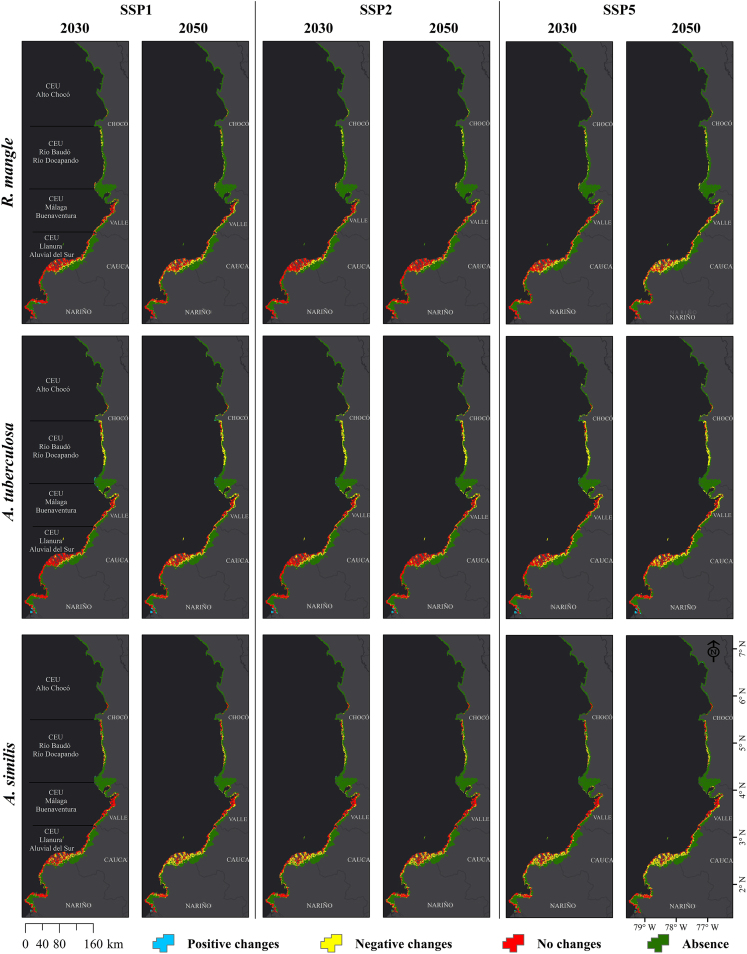
Changes in current and future environmental suitability (2030 and 2050, under climate scenarios SSP1, SSP2, and SSP5) for Piangua (*A. tuberculosa* and *A. similis*) and mangrove (*R. mangle*) in the Coastal Environmental Units of the Colombian Pacific

**Figure 7:**
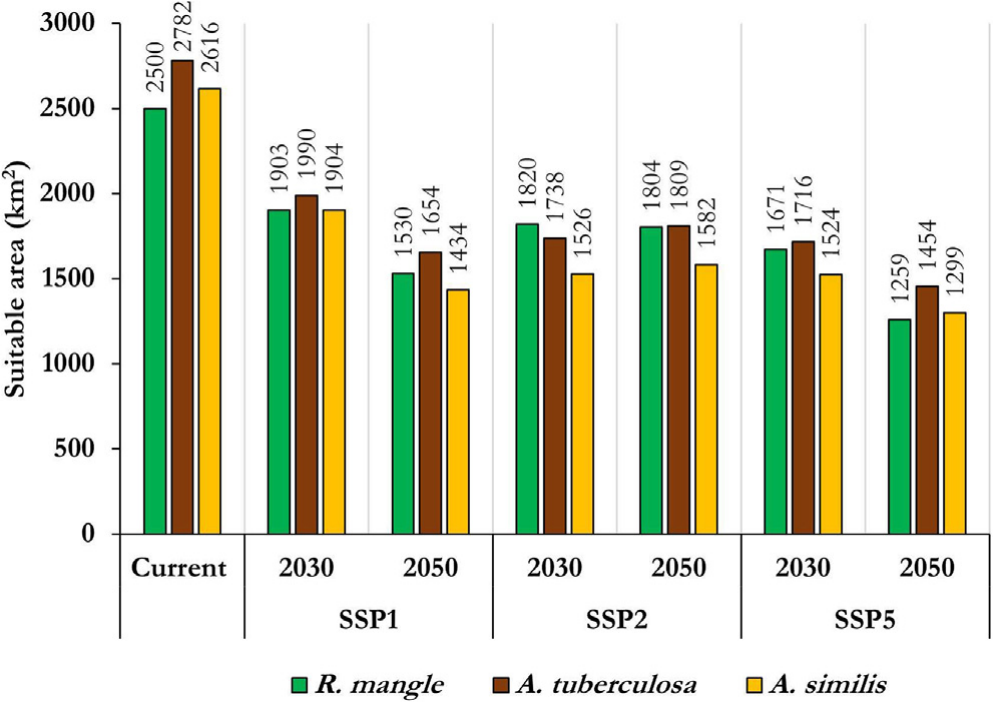
Quantification of current and future environmental suitability for potential distribution for Piangua (*A. tuberculosa* and *A. similis*) and mangrove (*R. mangle*) in the Coastal Environmental Units of the Colombian Pacific (7,328.78 km^2^)

Changes in environmental suitability for the Piangua (*A. tuberculosa* and *A. similis*) and mangrove (*R. mangle*) species in the CEUs of the Colombian Pacific are detailed in the supplemental information. This includes Negative changes, Positive changes, No changes, and Absence. For an overview covering the entire Pacific coast, refer to Table S2. Detailed breakdowns for each coastal department (Nariño, Cauca, Valle del Cauca, and Chocó) are provided in Tables S3–S6 and Figures S6–S9, respectively. The potential future species distribution in the study area varied spatially and temporally. Still, the potential distribution patterns of Piangua and mangrove tend to mirror those presented under current scenarios. Although future models reflect a similar spatial-temporal pattern to those under current scenarios, the environmental suitability of the species is impacted by climate change, particularly under the pessimistic climate change scenario (SSP5) (Figures 5 and 6). Figure 7 summarizes the potential distribution changes of the species in the study area, considering both current and future time periods (2030 and 2050) and the optimistic (SSP1), intermediate (SPP2), and pessimistic (SSP5) climate change scenarios. The current environmental suitability for mangrove *R. mangle*, *A. tuberculosa*, and *A. similis* was approximately 2,500; 2,782; and 2,616 km^2^, respectively, whereas for 2050 under the pessimistic scenario (SSP5), it was reduced to about 1,259; 1,454; and 1,299 km^2^, respectively.

Changes in environmental suitability for the species in the CEU related to the department of Nariño showed a trend consistent with what was observed for the entire Colombian Pacific (Figure S7). In this CEU, the potential future distribution area for *R. mangle* was generally larger than that projected for the Piangua, especially considering the area estimated for *A. similis*. Conversely, in the CEUs associated with the departments of Cauca, Valle del Cauca, and Chocó, the potential future distribution area was predominantly larger for the Piangua (particularly for *A. similis*) than for the mangrove (*R. mangle*). These trends might be attributable to *R. mangle’s* closer natural association with *A. tuberculosa* than with *A. similis*, as Mackenzie^2^ and Agudelo^1^ noted. Furthermore, the CEU related to the department of Nariño boasts the highest mangrove coverage and the densest population of *A. tuberculosa* in the Colombian Pacific, as documented by Delgado.^3^

The findings of Soon and Zheng^56^ highlight how climate change can negatively impact the distribution of the Piangua, underscoring the broader threat to bivalves from global warming. Similarly, Castro-Olivares^57^ discovered that in northeastern Spain, bivalves in shallower estuarine zones are affected by climate change, with the habitat of some species expected to diminish as water temperatures rise by the end of the century. On the Colombian Pacific coast, Borda and Cruz^10^ observed that climate change adversely impacts the availability and fishing of Piangua.

Environmental suitability projections for mangrove *R. mangle* align with Record,^21^ who reported that several mangrove species might lose suitable areas for their distribution, especially in Central America and the Caribbean. Also, Gouvêa^58^ predicted that, under a pessimistic climate change scenario (Representative Concentration Pathway [RCP; global radiation of 8.5 W/m^2^]), severe losses of suitable area for mangroves might occur, predominantly in tropical regions. In Colombia, Urrego^59^ reported that when precipitation decreases for longer than the regular dry season (e.g., during extreme El Niño events), mangrove forests may disappear or undergo species composition changes.

**Figure 8:**
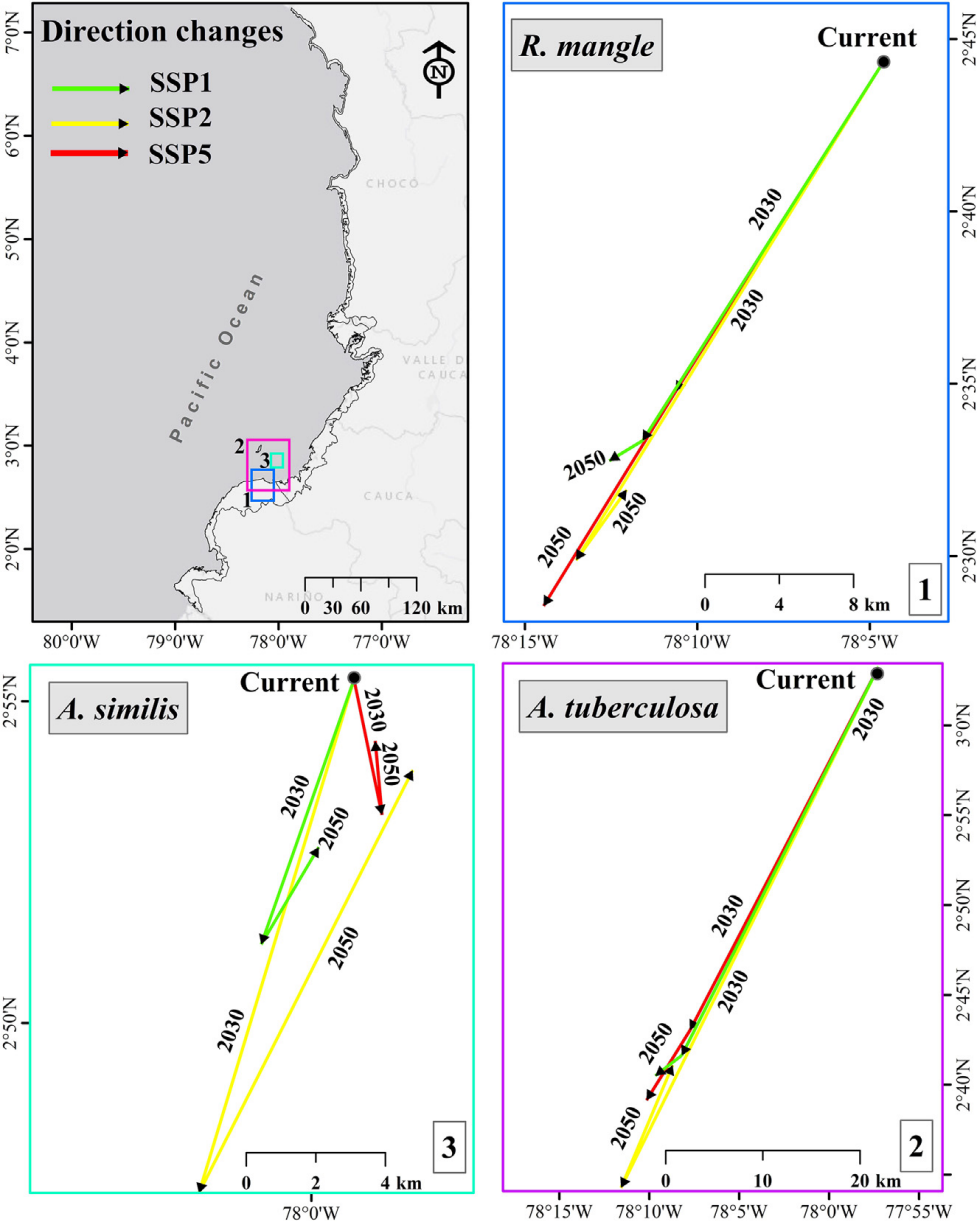
Changes in the direction of current and future environmental suitability (2030 and 2050, under climate scenarios SSP1, SSP2, and SSP5) for Piangua (*A. tuberculosa* and *A. similis*) and mangrove (*R. mangle*) in the Coastal Environmental Units of the Colombian Pacific

In general, the changes in the direction of environmental suitability of the modeled species vary among them. However, a similar pattern was identified between the projected potential future distribution of the mangrove *R. mangle* and Piangua, especially with the species *A. tuberculosa* (Figure 8). Under the future (2030 and 2050), Piangua (especially, *A. tuberculosa*) and mangrove (*R. mangle*) are predicted to be distributed more predominantly in a southwestern direction. However, by 2050 (especially for *A. similis*, in SSP2 scenario), slight changes in its potential distribution are expected, causing their environmental preference to shift north-easterly.

The directional shifts shown in Figure 8 are consistent with the changes in the potential future distribution of the species in the CEUs of the Colombian Pacific (Figures S6–S9). In the CEUs associated to the departments of Nariño, Cauca, and Valle del Cauca, it is evident that the potential future distribution of *R. mangle* aligns more closely with *A. tuberculosa* than with *A. similis*. In the CEU tied to Nariño, the potential distribution area for both *R. mangle* and *A. tuberculosa* was notably larger than that estimated for *A. similis*. In contrast, the preferred area for *R. mangle* and *A. tuberculosa* exhibited a declining trend (when compared against the area modeled for *A. similis*) in CEUs located northward in the Colombian Pacific, namely Cauca, Valle del Cauca, and Chocó. The patterns discerned between the potential distributions of *R. mangle* and *A. tuberculosa* align with their natural interrelationship, given that *A. tuberculosa* is frequently found within mangrove swamps dominated by *R. mangle*, as indicated by Mackenzie^2^ and Delgado.^3^ These findings may be related to the reduction in precipitation and the increase in temperature in the tropics, as some species, such as mangroves, tend to shift toward the poles, replacing the marshes that are located in the subtropical and warm temperate latitudinal limits.^21^^,^^46^^,^^60^^,^^61^ Rodríguez-Medina^41^ also reported that the environmental suitability of several mangrove species in Mexico is affected by climate change, as the suitable habitat for these species tends to shrink and shift toward the poles due to climate change. Some bivalve species are not exempt from experiencing these effects, as it has been predicted that several species (Bivalvia: *Solenidae*) may alter their distribution ranges toward the poles in response to future environmental changes.^62^ The identified changes in environmental suitability may generate spatial and temporal mismatches between the potential distribution of mangrove and Piangua species, as has been predicted for other species that maintain significant biological relationships with each other.^63^^,^^64^^,^^65^^,^^66^^,^^67^

The results of this study provide further evidence of the impacts climate change can have on mangroves and bivalves, such as the Piangua. The environmental suitability maps produced in this study can be a crucial tool for developing strategies to enhance the management and conservation of the Piangua and mangroves along the Colombian Pacific coast. These strategies could include zoning Piangua capture areas, establishing spatial fishing closures, rotating fishing areas, conserving fishing banks, creating protected areas, and developing a geographic information system to manage species occurrence, control, and conservation information. Such measures are supported by recommendations from official national entities^68^^,^^69^ and corroborated by studies conducted with fishing communities in the region.^3^^,^^8^ Models of the possible future distribution of the Piangua and the mangrove may be useful to strengthen continuous field monitoring of the species and track changes as they occur, providing a possible baseline for adaptive management in response to emerging circumstances and changing events such as climate change.^3^^,^^51^^,^^70^

### Limitations of the study

Direct physicochemical conditions of the ocean (e.g., salinity, sea temperature, ocean currents, etc.) were not considered in this study. Furthermore, the quality of the habitat was not considered, as well as the fishing pressure of the resource. These variables can affect the potential distribution of mangrove bivalves and therefore, the accuracy of predictive models. In this sense, other researchers are encouraged to strengthen the results presented here by building predictive models that incorporate these variables.

In this study, it was evident that the potential distribution of the Piangua varies spatially and temporally in the CEUs of the Colombian Pacific. Furthermore, it was identified that the potential distribution of these bivalves is influenced by the physicochemical conditions of the soil and bioclimatic variables that also vary spatiotemporally in the study area. Additional studies could evaluate Piangua distribution changes as a function of changes in environmental conditions on a finer temporal scale (e.g., monthly) within the study area.

### Conclusions

The application of machine learning models facilitated the identification of areas within the Colombian Pacific Coastal Environmental Units that could potentially host the Piangua (*A. tuberculosa* and *A. similis*) and the mangrove (*R. mangle*). The current environmental suitability for these species varied spatially within the study area. The current scenarios revealed that much of the coastal areas associated with the Nariño and Cauca CEUs, as well as the southern coastal regions of Valle del Cauca and Chocó showed a high probability for the distribution of mangroves and Piangua.

Climate change is anticipated to affect the environmental suitability of Piangua and mangrove. Future scenarios suggested that the spatial distribution of Piangua tends to follow similar patterns to those identified in the current scenarios. However, areas with 50 % (probability) or more environmental suitability may decrease due to climate change, especially by 2050 under the pessimistic climate change scenario (SSP5).

Under the future (2030 and 2050), the Piangua (especially *A. tuberculosa*) and the mangrove (*R. mangle*) predicted to be distributed with greater predominance in the southwestern direction of the Colombian Pacific coast. However, climate change can cause spatiotemporal mismatches between the potential distribution of these species (especially between *R. mangle* and *A. similis*) in some regions of the Colombian Pacific coast. These findings might be related to the reduction in precipitation and the increase in temperature in the tropics, as species such as mangroves tend to relocate toward the poles, replacing the marshes that are located in the subtropical and warm temperate latitudinal limits.

The results of this study provide valuable information for the management and conservation of species on the Colombian Pacific coast. Areas suitable for species' current and future distribution should be prioritized for conservation because they are expected to be more resilient to climate change over time. Conserving areas suitable for the current potential distribution of species is also crucial, as this helps safeguard species and prevent the loss of additional suitable habitats. Additionally, new sites of potential species distribution should be protected to address future climate change impacts. The conservation of these areas could mitigate the negative effects of climate change on species distribution.

## STAR★Methods

### Key resources table

**Table:** 

REAGENT or RESOURCE RESOURCE	SOURCE	IDENTIFIER
**Deposited data**		

Species occurrence data	Global Biodiversity Information Facility –GBIF	https://www.gbif.org/
Species occurrence data	Marine Environmental Information System –SiAM	https://siam.invemar.org.co/sibm-busqueda-avanzada
Bioclimatic variables	WorldClim (version 2.1)	https://www.worldclim.org/
Soil physicochemical variables	SoilGrids (version 2)	https://soilgrids.org/
Digital Elevation Model –DEM	Global Multi-Resolution Topography –GMRT	https://www.gmrt.org/GMRTMapTool/

**Software and algorithms**		

ArcGIS (version 10.8-1)	ESRI	https://desktop.arcgis.com/es/arcmap/latest/get-started/installation-guide/installing-on-your-computer.htm
R (version 4.2-3)	R Core Team	https://cran.r-project.org/bin/windows/base/old/4.2.3/
Biomod2 package (version 4.2-2)	Biomod2: Ensemble Platform for Species Distribution Modeling	https://cran.r-project.org/src/contrib/Archive/biomod2/
Raster package (version 3.6-20)	Raster: Geographic Data Analysis and Modeling	https://cran.r-project.org/src/contrib/Archive/raster/

### Resource availability

#### Lead contact

Further information and requests for resources should be directed to the lead contact, John Josephraj Selvaraj (jojselvaraj@unal.edu.co).

#### Materials availability

This study did not generate new unique reagents or materials.

#### Data and code availability

• All data reported in this paper will be shared by the lead contact upon request.
• This paper does not report original code.
• Any additional information required to reanalyze the data reported in this paper is available from the lead contact upon request.

### Method details

#### Study area

The study area encompasses the Coastal Environmental Units –CEU associated with the departments of Chocó, Valle del Cauca, Cauca, and Nariño in the Colombian Pacific. The CEUs included in this study are the following: (A) Upper Chocó, (B) Baudó River–Docapando River, (C) Málaga–Buenaventura, and (C) the Southern Alluvial Plain (Figure 7). Other studies have geographically delineated these areas based on distinct characteristics of the ecosystems they contain, as well as their structural and functional aspects.^71^ The Alto Chocó CEU stretches from the border with Panama to Cabo Corrientes in the department of Chocó. The Baudó River–Docapando River, starts from Cabo Corrientes and extends to the San Juan River delta in the Chocó department. Málaga–Buenaventura is bounded between the San Juan River delta and the Naya River's mouth in the Valle del Cauca department. Finally, the Southern Alluvial Plain spans from the mouth of the Naya River (Cauca) to the mouth of the Mataje River in the department of Nariño.^71^

The CEUs of the Colombian Pacific primarily consist of marine or littoral areas, including estuaries and marshes. Beaches, cliffs, and alluvial formations.^72^ The climatology of these areas diverges from other regions of the country, with an average annual precipitation between 3,000 and 12,000 mm, an average annual temperature between 24 and 28°C, an average relative humidity between 73 and 92%, and a terrain elevation ranging from sea level to 520 meters above sea level.^73^

The CEUs of the Colombian Pacific were selected for this study as they harbor the country’s most significant number of mangrove and Piangua species.^3^ These areas might encompass more than the current natural range of the species in question. However, their inclusion as a geographic boundary for the environmental suitability models was strategic, aiming to evaluate whether future environmental changes could enhance the ecological suitability of the species involved in this study. A similar survey by Rodriguez-Medina^41^ used a geographic delimiter larger than a species' natural habitat, utilizing a 35 km buffer along the Mexican Pacific coast to predict the environmental suitability of several mangrove species. Furthermore, this decision was supported by the assumption that all mangrove communities are within 30 km of the coast.^74^

#### Methodological process

Figure 8 shows a flow chart that summarizes the methodological process used for selecting data, choosing models, determining geographic distribution, and assessing changes in environmental suitability for *A. tuberculosa* and *A. similis*. This process considers climate change and the potential distribution of mangroves (*R. mangle*) in the Colombian Pacific coast.

#### Species selection and occurrence data

Colombia has a rich range of bivalves, with around 352 species in the Pacific Ocean and 315 in the Caribbean Sea.^75^^,^^76^ Out of the total species identified in the country, about 40 exhibit characteristics that render them commercially valuable, 18 of which are distributed in the Colombian Pacific coast. Among these, the Piangua is particularly notable for its abundance, recognition, and commercial importance.^77^ According to recent studies, the Piangua, the species *A. tuberculosa*, is the most heavily exploited mollusk in the Colombian Pacific coast.^78^ From January 16 and December 30, 2020, a total of 477.7 ton of mollusks were harvested, out of which 396.7 ton were of the Piangua (*A. tuberculosa* (370.7 ton) and *A. similis* (26 ton)) and the rest (81 ton) of other species (*Anadara spp*, *Melongena patula*, *Loliolopsis diomedeae* and *Dosidicus gigas*).^79^ In this context, the decision was made to model the environmental suitability of the Piangua due to its commercial significance, cultural relevance, and conservation needs in the Colombian Pacific coast.^3^

The spatial distribution of *A. tuberculosa* and *A. similis* is tied to the mangrove ecosystem, especially the species *R. mangle*.^2^ In this vein, the environmental suitability of the mangrove *R. mangle* in the study area was first modeled. Subsequently, the probabilistic layer of the potential distribution of the mangrove was used as one of the predictor variables to model the environmental suitability of the two species of Piangua.

The records of occurrences of the Piangua species were procured from biological record databases such as the Marine Environmental Information System –SiAM and Global Biodiversity Information Facility –GBIF.^80^^,^^81^^,^^82^ Species occurrence points were filtered based on the following criteria: No duplicates, presence of geographic coordinates (longitude and latitude) and falling within the study area. Additionally, records found within an area of 250 m^2^ (the spatial resolution of the environmental suitability models) were condensed into a single occurrence data point; this because the soil physicochemical variables (environmental predictors) were available at a spatial resolution of 250 m^2^ and SDMs based on machine learning only require an occurrence data to represent the value of a pixel of an environmental variable of interest.^83^^,^^84^

To model the environmental suitability of the mangrove *R. mangle*, 100 occurrence data points were artificially generated. It is assumed that *R. mangle* is the most abundant mangrove species on the Colombian Pacific coast,^1^ which justifies the procedure for generating the occurrence data of *R. mangle* in the study area. The occurrence data for *R. mangle* were randomly generated within the mangrove cover that has already been estimated for the Colombian Pacific.^85^

To enhance the prediction and validation of machine learning based SDMs, pseudo-absence data were introduced for the species being modeled. These artificially geolocated records denote areas unsuitable for potential species distribution.^22^^,^^86^ The confirmed absence data for species in specific locations are often scarce or non-existent, necessitating the random generation of pseudo-absence points.^86^ This was done with adherence to three criteria. The first criterion was to generate pseudo-absences in a 1:1 ratio relative to the actual occurrence data for the studied species.^86^ The second criterion was to create pseudo-absences outside the geographic limit associated with the identified mangrove cover for the Colombian Pacific coast.^85^ The third criterion required the generation of pseudo-absences within zones that contrasted with the environmental conditions in which the actual occurrences of the species were observed. To address the third criterion, the Surface Range Envelope –SRE model was used to classify the study area into potentially suitable and unsuitable zones. The SRE model incorporated the occurrence data for each species and the respective environmental variables that may influence their potential distribution. The SRE model was run using the Biomod2 package version 4.2-2, with a 0.025 quantile considered (corresponding to a 95% confidence interval). This approach was chosen to exclude the most extreme values of the environmental variables, thereby determining the limits of environmental suitability of the original occurrence locations.^87^

#### Selection of environmental variables

The current environmental suitability models for the Piangua species and the mangrove (*R. mangle*) incorporate the following predictor variables: Bioclimatic variables obtained from WorldClim version 2.1^88^ and soil physicochemical variables derived obtained from SoilGrids,^83^ Digital Elevation Model –DEM from the Global Multi-Resolution Topography –GMRT,^89^ and the Distance to Water Bodies –DWB calculated using the "Euclidean distance" tool in ArcGIS 10.8.1 software. Lastly, the potential distribution of the mangrove *R. mangle* (a probabilistic layer ranging from 0 [least suitable] and 1 [most suitable]) was integrated as a predictor variable to model the environmental suitability of the Piangua. The potential mangrove distribution was produced during this study.

The bioclimatic variables incorporated represent the monthly averages (1970 to 2000) of precipitation and temperature. These variables are commonly used in potential species distribution models due to their biological significance.^88^ The soil physicochemical variables are products of extensive studies over time.^83^ These variables have also been used to predict the potential distribution of species such as mangroves.^41^ The combined use of climatic and edaphic variables can enhance the accuracy of the SDMs.^90^ Table 2 concisely describes the environmental variables included in the environmental suitability models for the Piangua species and the mangrove (*R. mangle*).

The Piangua species are found at varying depths in the mangrove swamp soils. In this context, arithmetic averages of the soil's physicochemical variables were calculated based on the depth interval suitable for each species. These averages were as follows: Between 5 and 30 cm for *A. tuberculosa*; between 15 and 30 cm for *A. similis*; and between 0 and 200 cm for the *R. mangle*. The depth ranges for each species was determined with the support of other studies.^1^^,^^10^

Multicollinearity between environmental variables can inflate the variance of the regression parameters in a statistical regression model. However, this concern is alleviated when predictive models based on machine learning are utilized.^91^^,^^92^ These models consider variables separately, capturing non-linear relationships and interactions among them. Consequently, they have the capability to capture species response patterns in a more realistic and precise manner.^23^^,^^24^^,^^49^^,^^93^^,^^94^ Rodríguez-Medina^41^ included multiple correlated variables to model the potential distribution of mangroves along the coast of Mexico. Additionally, Braunisch,^35^ Harris,^36^ Porfirio,^37^ and Bucklin^95^ reported that constructing SDMs incorporating multiple correlated climate variables can be advantageous for modeling species distribution under future climate change, particularly in studies focusing on species conservation. Considering these findings and the limited information on the significance of environmental predictors in constructing models for predicting the potential distribution of the Piangua in the Colombian Pacific coast, it was decided to include the entire set of predictors (Table 2).

To evaluate the effect of climate change on the environmental suitability of the species, bioclimatic variables projected in the medium term (2030: Average between 2021 and 2040) and long term (2050: Average between 2041 and 2060) were included. These variables resulted from projections by the Global Circulation Model –GCM Max Planck Institute Earth System Model (MPI-ESM1-2-HR), which accurately predicts climate conditions in Colombia.^96^ The future projections contemplated three climate change scenarios: An optimistic scenario (Shared Socio-economic Pathways –SSP1), an intermediate (SSP2), and a pessimistic one (SSP5). These scenarios predict that by 2100, global warming could range between 3 to 3.5, 3.5 to 3.8, and 4.7°C to 5.1°C respectively,^16^ as described by the Intergovernmental Panel on Climate Change –IPCC in the sixth phase of the Coupled Model Intercomparison Project –CMIP6.^97^^,^^98^ The future projections of soil physicochemical variables as a climate change function were unavailable. Therefore, the same soil physicochemical variables used in the current models for each species were included in the future models, following the approach of other studies.^21^

The bioclimatic variables presented a different spatial resolution (1 km^2^) than the soil physicochemical variables (250 m^2^). Therefore, the pixel size of the bioclimatic predictors was adjusted to the finer resolution (250 m^2^). To reduce the spatial scale of the bioclimatic variables, the following steps were performed: Conversion of the raster layers (bioclimatic variables) to points. Random sampling of the points (70% for training and 30% for testing). Application of kriging type regression, including terrain elevation as a regressor for some variables (70% of the points). Validation of the downscaling results using the Mean Absolute Error –MAE, Root Mean Square Error –RMSE, and the R-Squared –Coefficient of Determination (30% of the data).^31^ The geoprocesses mentioned in the previous steps were executed with ArcGIS 10.8.1 software. Other authors have employed the proposed downscaling method (kriging type regression) to transform a coarse spatial resolution (10 km^2^) of bioclimatic variables into a finer one (700 m^2^).^99^

#### Selection of models, configurations, and evaluation parameters

The current and future environmental suitability of Piangua and mangrove species was modeled with algorithms based mainly on machine learning. Firstly, each of the following algorithms was run individually: General Boosting Model –GBM,^100^ Maximum Entropy Model –MaxEnt,^24^ Flexible Discriminant Analysis –FDA,^101^ Multiple Adaptive Regression Splines –MARS,^102^ Artificial Neuronal Network –ANN,^103^ Generalized Linear Model –GLM,^104^ Random Forest –RF,^105^ and Classification Tree Analysis –CTA.^106^ Each algorithm was run ten times with three permutations, considering the default settings in the Biomod2 package version 4.2-2,^107^ except for the model evaluation parameters, which included cross-validation with three partitions (k = 3) and ten repetitions. Cross-validation consisted of each subset of occurrence and pseudo-absence data being successively used for model validation, while the other k-1 subsets were used for calibration.^107^^,^^108^

The models built with the algorithm with the highest predictive performance in the calibration and validation stage were identified. Subsequently, the models with a predictive performance greater than 0.8 were ensembled (Weighted Mean Ensemble Model –EMwMean)^109^; this was determined based on the True Skill Statistic –TSS test.^109^^,^^110^ The final ensemble between SDMs was evaluated based on the KAPPA statistical tests,^111^ TSS,^109^^,^^110^ and AUC-ROC.^22^

To evaluate the effect of climate change on the environmental suitability of the species, we considered the differences in probability between the current and future potential distribution models. Changes in environmental suitability were quantified using methods proposed by Portilla-Cabrera & Selvaraj,^112^ which involve converting probabilistic maps of environmental suitability into binary maps indicating the presence and absence of species. The criterion for determining the presence or absence of a species in each pixel involved selecting the threshold that maximizes the sum of sensitivity and specificity (*maxSSS*). This optimal threshold is associated with the TSS test, as outlined by Liu,^113^^,^^114^ and was applied to each ensemble between the SDMs built for each species. The *maxSSS* values obtained in the SDMs of the species *A. tuberculosa*, *A. similis* and *R. mangle* were approximately 0.476, 0.501, and 0.572, respectively. Therefore, the cut-off point to transform the continuous maps to binary maps was set to 50% (probability).

Changes in environmental suitability were categorized as Negative changes, Positive changes, No changes, and Absence. Species distribution changes were discriminated by time period (2030 and 2050), climate scenario (SSP1, SSP2, and SSP5), and by CEU associated with each department of the Colombian Pacific (Nariño, Cauca, Valle del Cauca, and Chocó). To quantify the current potential distribution area of the species, the regions represented by the classes "Negative changes" and "No changes" were summed. Likewise, the regions defined by the classes "No changes" and "Positive changes" were added to quantify the potential future area. The processes to determine potential distribution changes of the species were performed with the R "raster" package.^115^ Furthermore, the direction of potential spatial distribution changes of the species were determined^116^; this was to identify possible trends in the geographic distribution of Piangua and mangrove (*R. mangle*) species.

Lastly, we determined the importance of the environmental variables included in SDMs. Identifying the most important environmental variables can help to understand the capability of these variables to discriminate the potential distribution of species with the proposed models. Additionally, environmental response curves were created, which related the values of the environmental variables (x-axis) as a function of the probability of occurrence of the species (y-axis). The importance of the environmental variables, as well as the environmental response curves, were obtained with the Biomod2 package version 4.2-2.

### Quantification and statistical analysis

Quantification and statistical analysis are presented in method details.

## References

[1] Agudelo D., Gualteros W., Delgado M.F., Lucero C.H., Espinosa S., Cortés N., Palacio C.J., Roldán A.M., Zapata L.A., Candelo C. (2010). Productive potential of the natural populations of the piangua *Anadara tuberculosa* and *Anadara similis* within a space-time perspective on the Colombian Pacific coast [Potencial productivo de las poblaciones naturales de la piangua *Anadara tuberculosa* y *Anadara similis* dentro de una perspectiva espacio-temporal en la costa Pacífica colombiana]. https://docplayer.es/79932769-Informe-final-contrato-t-diciembre-9-de-vinculado-al-ministerio-de-ambiente-vivienda-y-desarrollo-territorial.html.

[2] MacKenzie C. (2001). The Fisheries for Mangrove Cockles, Anadara spp., from Mexico to Peru, with Descriptions of Their Habitats and Biology, the Fishermen’s Lives, and the Effects of Shrimp Farming. https://aquadocs.org/bitstream/handle/1834/26374/mfr6311.pdf?sequence=1&isAllowed=y.

[3] Delgado M.F., Walteros W., Espinosa S., Lucero C., Roldan A.M., Zapata L.A., Cantera J.R., Candelo C., Palacio C., Muñoz O. (2010). Pianguando - Strategies for the management of the Piangua [Pianguando - Estrategias para el manejo de la piangua]. http://hdl.handle.net/1834/8253.

[4] Devis-Morales A., García-Hansen I., Málikov I., Villegas-Bolaños N.L. (2002). Oceanographic compilation of the Colombian Pacific basin [Compilación oceanográfica de la cuenca Pacífica colombiana]. https://www.academia.edu/38744126/Compilacin_Oceanogrfica_de_la_Cuenca_Pacfica_Colombiana.

[5] García C. (2010). Diagnosis of marine and coastal protected areas, and management areas in the Colombian Pacific [Diagnóstico de las áreas marinas y costeras protegidas, y de las áreas de manejo en el Pacífico colombiano]. https://marviva.net/wp-content/uploads/2021/11/amp_colombia.pdf.

[6] Abud M., Andrade A., Guevara O., Herrera-Carmona J., Pavón H., Rodríguez E., Zapata-Padilla L. (2014). Climate change and vulnerability in marine and coastal areas and adaptation strategies of the Colombian Pacific [Cambio climático y vulnerabilidad, en áreas marinas y costeras y estrategias de adaptación del Pacífico colombiano]. http://www.invemar.org.co/documents/67592/87372/CA-12.pdf/5a280caa-f762-4ad7-8daa-862f921ac85b.

[7] Herrera-Carmona J.C., Zapata-Padilla L.A., Moreno-Gutiérrez X. (2014). Vulnerability, climate change and adaptation strategies in marine and coastal areas of the Colombian Pacific [Vulnerabilidad, cambio climático y estrategias de adaptación en áreas marinas y costeras del Pacífico colombiano]. https://www.researchgate.net/publication/270591382_Vulnerabilidad_cambio_climatico_y_estrategias_de_adaptacion_en_areas_marinas_y_costeras_del_Pacifico_colombiano.

[8] Portilla-Cabrera C.V., Cifuentes-Ossa M.A., Selvaraj J.J. (2024). Policy-oriented adaptation strategies for artisanal marine fisheries under climate and non-climate stressors in San Andres de Tumaco, Nariño, Colombia. Mar. Policy.

[9] Espinosa-Guerrero S. (2011). Large-scale spatial variation of the density and size of Anadara tuberculosa (Sowerby, 1833) as basic information for the management of this economically important species on the Colombian Pacific coast [Variación espacial a gran escala de la densidad y el tamaño de Anadara tuberculosa (Sowerby, 1833) como información base para el manejo de esta especie de importancia económica, en la costa del pacífico colombiano]. http://www.invemar.org.co/redcostera1/invemar/docs/RinconLiterario/2012/abril/CD-263_T-711.pdf.

[10] Borda C.A., Cruz R. (2004). Artisanal bivalve fishing (*Anadara tuberculosa* and *A. similis*) and its relationship with environmental events. Colombian Pacific [Pesca artesanal de bivalvos (*Anadara tuberculosa* y *A. similis*) y su relación con eventos ambientales. Pacífico colombiano]. Rev. Invest..

[11] Ardila N., Navas G., Reyes J. (2002). Red book of marine invertebrates of Colombia [Libro rojo de invertebrados marinos de Colombia]. http://www.invemar.org.co/redcostera1/invemar/docs/lrojo/LR_INVERTEBRADOS.pdf.

[12] Velasco L.C., Chacón E.M.A., Ardila N., Pérez G.H.B., Campos N.H., Quintero K.J.M. (2022). Red book marine vertebrates of Colombia (2022). Red Book of Invertebrates [Libro rojo vertebrados marinos de Colombia (2022). Libro rojo de invertebrados], 1–394. https://www.researchgate.net/publication/366481398_Libro_Rojo_de_Invertebrados_Marinos_de_Colombia_2022.

[13] Laffoley D., Baxter J.M. (2016).

[14] Pachauri R.K., Meyer L., Allen M.R., Barros V.R., Broome J., Cramer W., Jiang K., Jiménez Cisneros México B., Kattsov V., Lee H. (2014). Climate change 2014 Synthesis report [Cambio climático 2014 Informe de síntesis]. https://www.ipcc.ch/site/assets/uploads/2018/02/SYR_AR5_FINAL_full_es.pdf.

[15] Ward R.D., Friess D.A., Day R.H., MacKenzie R.A. (2016). Impacts of climate change on mangrove ecosystems: a region by region overview. Ecosyst. Health Sustain..

[16] Hausfather Z. (2018). Explainer: How ‘Shared Socioeconomic Pathways’ Explore Future Climate Change - Carbon Brief. https://www.carbonbrief.org/explainer-how-shared-socioeconomic-pathways-explore-future-climate-change/.

[17] Field C.B., Barros V.R., Dokken D.J., Mach K.J., Mastrandrea Codirector M.D., Calvo Buendía E., Moreno J.M., Eren Bilir T., Chatterjee M., Yuka K.L.E. (2014). Climate Change 2014 Impacts, Adaptation and Vulnerability [Cambio Climático 2014 Impactos, Adaptación Y Vulnerabilidad]. https://www.ipcc.ch/site/assets/uploads/2018/03/ar5_wgII_spm_es-1.pdf.

[18] Cruz R., Borda C. (2003). Exploitation status and forecast of the *Anadara tuberculosa* fishery (Sowerby, 1833) in the Colombian Pacific [Estado de explotación y pronóstico de la pesquería de *Anadara tuberculosa* (Sowerby, 1833) en el Pacífico Colombiano]. Rev. Investig. Mar..

[19] Lucero C., Cantera J., Neira R. (2012). Fishery and growth of the piangua (Arcoida: *Arcidae) Anadara tuberculosa* in the Bay of Malaga of the Colombian Pacific, 2005-2007 [Pesquería y crecimiento de la piangua (Arcoida: *Arcidae) Anadara tuberculosa* en la Bahía de Málaga del Pacífico colombiano, 2005-2007]. Rev. Biol. Trop..

[20] Lucero-Rincón C.H., Cantera K. J.R., Gil-Agudelo D.L., Muñoz O., Zapata L.A., Cortes N., Gualteros W.O., Manjarres A., Manjarres A. (2013). Spatiotemporal analysis of the reproductive biology and recruitment of the bivalve mollusk *Anadara tuberculosa* on the Colombian Pacific coast [Análisis espacio temporal de la biología reproductiva y el reclutamiento del molusco bivalvo *Anadara tuberculosa* en la costa del Pacífico colombiano]. Rev. Biol. Mar. Oceanogr..

[21] Record S., Charney N.D., Zakaria R.M., Ellison A.M. (2013). Projecting global mangrove species and community distributions under climate change. Ecosphere.

[22] Elith J., Graham C., Anderson R., Dudík M., Ferrier S., Guisan A., Hijmans R., Huettmann F., R. Leathwick J., Lehmann A. (2006). Novel methods improve prediction of species’ distributions from occurrence data. Ecography.

[23] Elith J., Leathwick J.R. (2009). Species Distribution Models: Ecological Explanation and Prediction Across Space and Time. Annu. Rev. Ecol. Evol. Syst..

[24] Phillips S., Anderson R., Schapire R. (2006). Maximum entropy modeling of species geographic distributions. Ecol. Modell..

[25] Miller J. (2010). Species Distribution Modeling. Geogr. Compass.

[26] Biodiversity and Climate Change Virtual Lab –BCCVL (2019). Overview of SDM Methods in BCCVL: BCCVL. https://support.bccvl.org.au/support/solutions/articles/6000083199-overview-of-sdm-methods-in-bccvl.

[27] Wiley E.O., McNyset K.M., Peterson A.T., Robins C.R., Stewart A.M. (2003). Niche Modeling Perspective on Geographic Range Predictions in the Marine Environment Using a Machine-learning Algorithm. Oceanography.

[28] Zhang L., Huettmann F., Liu S., Sun P., Yu Z., Zhang X., Mi C. (2019). Classification and regression with random forests as a standard method for presence-only data SDMs: A future conservation example using China tree species. Ecol Inform.

[29] Beery S., Cole E., Parker J., Perona P., Winner K. (2021). Proceedings of the 4th ACM SIGCAS Conference on Computing and Sustainable Societies.

[30] Chollet-Ramampiandra E., Scheidegger A., Wydler J., Schuwirth N. (2023). A comparison of machine learning and statistical species distribution models: Quantifying overfitting supports model interpretation. Ecol. Modell..

[31] Njeban H.S. (2018). Comparison and Evaluation of GIS-Based Spatial Interpolation Methods for Estimation Groundwater Level in AL-Salman District—Southwest Iraq. J. Geogr. Inf. Syst..

[32] Maravillas A.B., Feliscuzo L.S., Nogra J.A.E. (2023). ACM International Conference Proceeding Series.

[33] Huang D., An Q., Huang S., Tan G., Quan H., Chen Y., Zhou J., Liao H. (2023). Biomod2 modeling for predicting the potential ecological distribution of three *Fritillaria* species under climate change. Sci. Rep..

[34] Grenouillet G., Buisson L., Casajus N., Lek S. (2011). Ensemble modelling of species distribution: the effects of geographical and environmental ranges. Ecography.

[35] Braunisch V., Coppes J., Arlettaz R., Suchant R., Schmid H., Bollmann K. (2013). Selecting from correlated climate variables: a major source of uncertainty for predicting species distributions under climate change. Ecography.

[36] Harris R.M.B., Porfirio L.L., Hugh S., Lee G., Bindoff N.L., Mackey B., Beeton N.J. (2013). To Be Or Not to Be? Variable selection can change the projected fate of a threatened species under future climate. Eco. Manag. Restor..

[37] Porfirio L.L., Harris R.M.B., Lefroy E.C., Hugh S., Gould S.F., Lee G., Bindoff N.L., Mackey B. (2014). Improving the Use of Species Distribution Models in Conservation Planning and Management under Climate Change. PLoS One.

[38] Liang W., Papeş M., Tran L., Grant J., Washington-Allen R., Stewart S., Wiggins G. (2018). The effect of pseudo-absence selection method on transferability of species distribution models in the context of non-adaptive niche shift. Ecol. Modell..

[39] Moreno-Martínez A., Álvarez-Arteaga G., Orozco-Hernández M.E. (2021). Heterogeneidad ambiental y alteraciones antrópicas en comunidades de manglar en el pacífico sur de México. Rev. Ambientales.

[40] Arias-Morán A.E., Castro-Molina K.V. (2017). https://www.dspace.espol.edu.ec/retrieve/129790/D-76626.pdf.

[41] Rodríguez-Medina K., Yañez-Arenas C., Peterson A.T., Ávila J.E., Herrera-Silveira J. (2020). Evaluating the capacity of species distribution modeling to predict the geographic distribution of the mangrove community in Mexico. PLoS One.

[42] Prado-Carpio E., Martinez-Soto M., Rodríguez-Monroy C., Quiñonez-Cabeza M., Olivo-Garrido M. (2021). Biology, productivity and commercial attributes of the bivalve mollusk «concha prieta» (*Anadara tuberculosa*) [Biología, productividad y atributos comerciales del molusco bivalvo «concha prieta» (*Anadara tuberculosa*)]. https://revistaespacios.com/a21v42n22/a21v42n22p02.pdf.

[43] Austin M.P. (2002). Spatial prediction of species distribution: an interface between ecological theory and statistical modelling. Ecol. Modell..

[44] Tchantse V., Cabrera-Luna E. (1998). Some research aspects of the formation of the oceanographic regime in the Colombian Pacific [Algunos aspectos de investigación de la formación del régimen oceanográfico en el Pacífico colombiano]. https://cecoldodigital.dimar.mil.co/369/1/dimarcccp_1998_boletincccp_07_07-19OK.pdf.

[45] Trojer H. (2017). Meteorología y climatología de la vertiente del Pacífico colombiano. Rev. Acad. Colomb. Cienc. Ex. Fis. Nat..

[46] Cavanaugh K.C., Kellner J.R., Forde A.J., Gruner D.S., Parker J.D., Rodriguez W., Feller I.C. (2014). Poleward expansion of mangroves is a threshold response to decreased frequency of extreme cold events. Proc. Natl. Acad. Sci. USA.

[47] Soberón J., Peterson A.T. (2005). Interpretation of Models of Fundamental Ecological Niches and Species’ Distributional Areas. Biodivers. Inf..

[48] Maciel-Mata C.A., Manríquez-Morán N., Octavio-Aguilar P., Sánchez-Rojas G. (2015). The distribution area of species: review of the concept [El área de distribución de las especies: revisión del concepto]. Acta Univ..

[49] Peterson A.T., Soberón J., Pearson R.G., Anderson R.P., Martínez-Meyer E., Nakamura M., Araújo M.B. (2011).

[50] Bernal B., Sidman G., Pearson T. (2017). Assessment of Mangrove Ecosystems in Colombia and Their Potential for Emissions Reductions and Restoration. https://winrock.org/wp-content/uploads/2018/02/6.-Coastal-assessment-in-Colombia.pdf.

[51] Rodríguez-Rodríguez J.A., Sierra-Correa P.C., Gómez-Cubillos M.C., Villanueva L.V.L. (2018).

[52] Murillo-Sandoval P.J., Fatoyinbo L., Simard M. (2022). Mangroves Cover Change Trajectories 1984-2020: The Gradual Decrease of Mangroves in Colombia. Front. Mar. Sci..

[53] Ramírez-Ochoa L.F. (2005). Factors affecting the propagation and establishment of *Avicennia germinans L*. in degraded environments of subtropical semiarid regions [Factores que afectan la propagación y establecimiento de *Avicennia germinans L*. en ambientes degradados de regiones semiáridas subtropicales]. https://hdl.handle.net/20.500.11801/1650.

[54] Gagnon K., Rinde E., Bengil E.G.T., Carugati L., Christianen M.J.A., Danovaro R., Gambi C., Govers L.L., Kipson S., Meysick L. (2020). Facilitating foundation species: The potential for plant–bivalve interactions to improve habitat restoration success. J. Appl. Ecol..

[55] Franco-Vidal L. (1995). Use and conservation of molluscs of the genus Anadara (Mollusca: Bivalvia)-population evidence in a gradient of human exploitation in Chocó, Colombian Pacific Coast [Uso y conservación de moluscos del género *Anadara* (Mollusca: Bivalvia)-evidencia poblacional en un gradiente de explotación humana en el Chocó, Costa Pacífica colombiana]. http://koha.ideam.gov.co/cgi-bin/koha/opac-detail.pl?biblionumber=4427603&shelfbrowse_itemnumber=.

[56] Soon T.K., Zheng H. (2020). Climate Change and Bivalve Mass Mortality in Temperate Regions. Rev. Environ. Contam. Toxicol..

[57] Castro-Olivares A., Des M., Olabarria C., deCastro M., Vázquez E., Sousa M.C., Gómez-Gesteira M. (2022). Does global warming threaten small-scale bivalve fisheries in NW Spain?. Mar. Environ. Res..

[58] Gouvêa L.P., Serrão E.A., Cavanaugh K., Gurgel C.F.D., Horta P.A., Assis J. (2022). Global impacts of projected climate changes on the extent and aboveground biomass of mangrove forests. Divers. Distrib..

[59] Urrego L.E., Correa-Metrio A., González-Arango C. (2018). Colombian Caribbean mangrove dynamics: anthropogenic and environmental drivers. Bol. Soc. Geol. Mex..

[60] Saintilan N., Wilson N.C., Rogers K., Rajkaran A., Krauss K.W. (2014). Mangrove expansion and salt marsh decline at mangrove poleward limits. Glob. Chang. Biol..

[61] Friess D.A., Adame M.F., Adams J.B., Lovelock C.E. (2022). Mangrove forests under climate change in a 2°C world. Wiley Interdiscip. Rev. Clim. Change.

[62] Saeedi H., Basher Z., Costello M.J. (2017). Modelling present and future global distributions of razor clams (Bivalvia: *Solenidae*). Helgol. Mar. Res..

[63] Schweiger O., Settele J., Kudrna O., Klotz S., Kühn I. (2008). Climate change can cause spatial mismatch of trophically interacting species. Ecology.

[64] Valladares F., Bastias C.C., Godoy O., Granda E., Escudero A. (2015). Species coexistence in a changing world. Front. Plant Sci..

[65] Parejo D. (2016). Informational mismatches: A neglected threat of climate change to interspecific interactions. Front. Ecol. Evol..

[66] Polce C., Garratt M.P., Termansen M., Ramirez-Villegas J., Challinor A.J., Lappage M.G., Boatman N.D., Crowe A., Endalew A.M., Potts S.G. (2014). Climate-driven spatial mismatches between British orchards and their pollinators: increased risks of pollination deficits. Glob. Chang. Biol..

[67] Pagel J., Treurnicht M., Bond W.J., Kraaij T., Nottebrock H., Schutte-Vlok A.L., Tonnabel J., Esler K.J., Schurr F.M. (2020). Mismatches between demographic niches and geographic distributions are strongest in poorly dispersed and highly persistent plant species. Proc. Natl. Acad. Sci. USA.

[68] Ministry of Environment and Sustainable Development –MADS (2018). Resolution 1263 of July 1, 2018 "Whereby measures to ensure the sustainability and comprehensive management of mangrove ecosystems are updated, and other determinations are made" [Resolución 1263 del 1 de julio de 2018 “Por medio de la cual se actualizan las medidas para garantizar la sostenibilidad y la gestión integral de los ecosistemas de manglar, y se toman otras determinaciones.”]. https://www.andi.com.co/Uploads/RES%201263.pdf.

[69] Ministry of Environment and Sustainable Development –MADS, World Wide Fund for Nature –WWF (2023). Update program for the sustainable use, management and conservation of mangrove ecosystems in Colombia [Actualización programa para el uso sostenible manejo y conservación de los ecosistemas de manglar en Colombia]. https://www.minambiente.gov.co/consulta/actualizacion-programa-para-el-uso-sostenible-manejo-y-conservacion-de-los-ecosistemas-de-manglar-en-colombia/.

[70] Rodríguez-Rodríguez J.A., Mancera-Pineda J.E., Tavera H. (2021). Mangrove restoration in Colombia: Trends and lessons learned. For. Ecol. Manage..

[71] Ministry of Environment and Sustainable Development –MADS (2013). Decree 1120 of 2013 "By which the Coastal Environmental Units (CEU) and the joint commissions are regulated, the rules of procedure and criteria are established to regulate the restriction of certain activities in seagrasses, and other provisions are issued" [Decreto 1120 de 2013 “Por el cual se reglamentan las Unidades Ambientales Costeras (UAC) y las comisiones conjuntas, se establecen las reglas de procedimiento y criterios para reglamentar la restricción de ciertas actividades en pastos marinos, y se dictan otras disposiciones”]. https://www.minambiente.gov.co/wp-content/uploads/2022/01/decreto-1120-de-2013.pdf.

[72] Avella F., Osorio A., Parra E., Burgos S., Vilardy S., Botero C., Ramos A., Mendoza J., Sierra P., López Á. (2018). Coastal management in Colombia. Challenge of a country with three coasts [Gestión del litoral en Colombia. Reto de un país con tres costas]. https://cco.gov.co/docs/ibermar/g_litoral.pdf.

[73] Institute of Hydrology Meteorology and Environmental Studies –IDEAM (2005). Part II spatiotemporal distribution of climate variables [Parte II distribución espacio-temporal de las variables del clima]. http://documentacion.ideam.gov.co/openbiblio/bvirtual/019711/AtlasClimatico2.pdf.

[74] Batllori-Sampedro E., Febles-Patrón J.L. (2007). Maximum permissible limits for the use of the mangrove ecosystem [Límites máximos permisibles para el aprovechamiento del ecosistema de manglar]. https://www.redalyc.org/pdf/539/53908201.pdf.

[75] Arboleda E. (2002). Current state of knowledge and richness of fish, crustaceans, decapods, mollusks, echinoderms and scleractinian corals of the Colombian Pacific Ocean [Estado actual del conocimiento y riqueza de peces, crustáceos, decápodos, moluscos, equinodermos y corales escleractinios del océano pacífico colombiano]. http://centrodocumentacion.invemar.org.co/cgi-bin/koha/opac-detail.pl?biblionumber=3465&query_desc=an%3A62.

[76] Díaz J.M., Carlos M., Betancourt V., Saldarriaga G.M. (2011). Diagnosis of the main fisheries of the Colombian Pacific [Diagnóstico de las principales pesquerías del Pacífico colombiano]. https://marviva.net/wp-content/uploads/2021/11/pesquerias_baja.pdf.

[77] Velasco L.A., Barros J. (2008). Cultivation of bivalves in Colombia: utopia or commitment to the future? [Cultivo de bivalvos en Colombia: ¿utopía o apuesta de futuro?]. https://www.researchgate.net/publication/316017630_Cultivo_de_bivalvos_en_Colombia_utopia_o_apuesta_de_futuro.

[78] Usma-Oviedo J.S., Rodríguez T., Moreno X., Franco-Jaramillo M., García-Llano C.F., Castellanos-Galindo G. (2020). Fishing resources of Colombia, main species, conservation and responsible fishing [Recursos pesqueros de Colombia, principales especies, conservación y pesca responsable]. https://wwflac.awsassets.panda.org/downloads/recursos_pesqueros_de_colombia.pdf.

[79] Duarte L.O., Cuervo C., Vargas O., Gil-Manrique B., Cuello F., De León G., Isaza-Toro E., Tejeda K., Manjarrés-Martínez L., Reyes-Ardila H. (2020). Landing and effort statistics of Colombia's artisanal fisheries during 2020 [Estadísticas de desembarco y esfuerzo de las pesquerías artesanales de Colombia durante el año 2020]. http://sepec.aunap.gov.co/Archivos/Boletines-2020/SEPEC_Boletin_Pesca_Artesanal_2020.pdf.

[80] Global Biodiversity Information Facility –GBIF (2023).

[81] Global Biodiversity Information Facility –GBIF (2023).

[82] Marine and Coastal Research Institute –INVEMAR (2023). https://siam.invemar.org.co/sibm-busqueda-avanzada.

[83] Hengl T., De Jesus J.M., Heuvelink G.B.M., Gonzalez M.R., Kilibarda M., Blagotić A., Shangguan W., Wright M.N., Geng X., Bauer-Marschallinger B. (2017). SoilGrids250m: Global gridded soil information based on machine learning. PLoS One.

[84] Phillips S. (2010). A Brief Tutorial on Maxent. https://biodiversityinformatics.amnh.org/open_source/maxent/Maxent_tutorial2017.pdf.

[85] Selvaraj J.J., Gallego-Pérez B.E. (2023). An enhanced approach to mangrove forest analysis in the Colombian Pacific coast using optical and SAR data in Google Earth Engine. Remote Sens. Appl..

[86] Barbet-Massin M., Jiguet F., Albert C.H., Thuiller W. (2012). Selecting pseudo-absences for species distribution models: how, where and how many?. Methods Ecol. Evol..

[87] Thuiller W., Lafourcade B., Engler R., Araújo M.B. (2009). BIOMOD - A platform for ensemble forecasting of species distributions. Ecography.

[88] Fick S.E., Hijmans R.J. (2017). WorldClim 2: new 1-km spatial resolution climate surfaces for global land areas. Int. J. Climatol..

[89] Ryan W.B.F., Carbotte S.M., Coplan J.O., O’Hara S., Melkonian A., Arko R., Weissel R.A., Ferrini V., Goodwillie A., Nitsche F. (2009). Global Multi-Resolution Topography synthesis. Geochem. Geophys. Geosyst..

[90] Velazco S.J.E., Galvão F., Villalobos F., De Marco P. (2017). Using worldwide edaphic data to model plant species niches: An assessment at a continental extent. PLoS One.

[91] Hastie T., Tibshirani R., Friedman J. (2009).

[92] Dormann C.F., Elith J., Bacher S., Buchmann C., Carl G., Carré G., Marquéz J.R.G., Gruber B., Lafourcade B., Leitão P.J. (2013). Collinearity: a review of methods to deal with it and a simulation study evaluating their performance. Ecography.

[93] Araújo M.B., Pearson R.G., Thuiller W., Erhard M. (2005). Validation of species–climate impact models under climate change. Glob. Chang. Biol..

[94] Phillips S.J., Dudík M. (2008). Modeling of species distributions with Maxent: new extensions and a comprehensive evaluation. Ecography.

[95] Bucklin D.N., Basille M., Benscoter A.M., Brandt L.A., Mazzotti F.J., Romañach S.S., Speroterra C., Watling J.I. (2015). Comparing species distribution models constructed with different subsets of environmental predictors. Divers. Distrib..

[96] Arias P.A., Ortega G., Villegas L.D., Martínez J.A. (2021). Colombian climatology in CMIP5/CMIP6 models: Persistent biases and improvements. Rev. Fac. Ing. Univ. Antioquia.

[97] Eyring V., Bony S., Meehl G.A., Senior C.A., Stevens B., Stouffer R.J., Taylor K.E. (2016). Overview of the Coupled Model Intercomparison Project Phase 6 (CMIP6) experimental design and organization. Geosci. Model Dev. (GMD).

[98] Riahi K., van Vuuren D.P., Kriegler E., Edmonds J., O’Neill B.C., Fujimori S., Bauer N., Calvin K., Dellink R., Fricko O. (2017). The Shared Socioeconomic Pathways and their energy, land use, and greenhouse gas emissions implications: An overview. Global Environ. Change.

[99] Somodi I., Molnár Z., Czúcz B., Bede-Fazekas Á., Bölöni J., Pásztor L., Laborczi A., Zimmermann N.E. (2017). Implementation and application of multiple potential natural vegetation models – a case study of Hungary. J. Veg. Sci..

[100] Elith J., Leathwick J.R., Hastie T. (2008). A working guide to boosted regression trees. J. Anim. Ecol..

[101] Hastie T., Tibshirani R., Buja A. (1994). Flexible Discriminant Analysis by Optimal Scoring. J. Am. Stat. Assoc..

[102] Leathwick J.R., Elith J., Hastie T. (2006). Comparative performance of generalized additive models and multivariate adaptive regression splines for statistical modelling of species distributions. Ecol. Modell..

[103] Hilbert D.W., Ostendorf B. (2001). The utility of artificial neural networks for modelling the distribution of vegetation in past, present and future climates. Ecol. Modell..

[104] Guisan A., Edwards T.C., Hastie T. (2002). Generalized linear and generalized additive models in studies of species distributions: Setting the scene. Ecol. Modell..

[105] Prasad A.M., Iverson L.R., Liaw A. (2006). Newer classification and regression tree techniques: Bagging and random forests for ecological prediction. Ecosystems.

[106] Thuiller W., Araújo M., Lavorel S. (2003). Generalized Models vs. Classification Tree Analysis: Predicting Spatial Distributions of Plant Species at Different Scales. https://www.jstor.org/stable/3236943.

[107] Thuiller W., Georges D., Gueguen M., Engler R., Breiner F., Lafourcade B., Patin R. (2023). https://cran.r-project.org/web/packages/biomod2/biomod2.pdf.

[108] Jung Y., Hu J. (2015). A K-fold Averaging Cross-validation Procedure. J. Nonparametr. Stat..

[109] Hao T., Elith J., Guillera-Arroita G., Lahoz-Monfort J.J. (2019). A review of evidence about use and performance of species distribution modelling ensembles like BIOMOD. Divers. Distrib..

[110] Allouche O., Tsoar A., Kadmon R. (2006). Assessing the accuracy of species distribution models: Prevalence, kappa and the true skill statistic (TSS). J. Appl. Ecol..

[111] Monserud R.A., Leemans R. (1992). Comparing global vegetation maps with the Kappa statistic. Ecol. Modell..

[112] Portilla-Cabrera C.V., Selvaraj J.J. (2020). Geographic shifts in the bioclimatic suitability for *Aedes aegypti* under climate change scenarios in Colombia. Heliyon.

[113] Liu C., White M., Newell G. (2013). Selecting thresholds for the prediction of species occurrence with presence-only data. J. Biogeogr..

[114] Liu C., Newell G., White M. (2016). On the selection of thresholds for predicting species occurrence with presence-only data. Ecol. Evol..

[115] Hijmans R.J. (2023). https://cran.r-project.org/web/packages/raster/raster.pdf.

[116] Brown J.L. (2014). SDMtoolbox: A python-based GIS toolkit for landscape genetic, biogeographic and species distribution model analyses. Methods Ecol. Evol..

