# Supplementary material for: Impact of climate change on Colombian Pacific coast mangrove bivalves distribution
Source: iScience. 2024 Jul 10;27(8):110473. doi: 10.1016/j.isci.2024.110473 (PMC11321327; doi:10.1016/j.isci.2024.110473)
Supplement: Document S1. Figures S1–S9 and Tables S1–S6 [file mmc1.pdf]

iScience, Volume *27*

Supplemental information

Impact of climate change on Colombian Pacific coast mangrove bivalves distribution

John Josephraj Selvaraj and Cristiam Victoriano Portilla-Cabrera

Supplementary information

Supplementary Figure S1, p. 1

Supplementary Figures S2 and S3, p. 2

>Cetuximab heavy chain(pepsin pH>2 cleavage fragment)

QVQLKQSGPGLVQPSQSLSIT**C**TVSGFSLTNYGVHWVRQSPGKGLEWLGVIWSGGNTDYN
TPFTSRLSINKDNSKSQVFFKMNSLQSNDTAIYY**C**ARALTYYDYEFAYWGQGTLVTVSAA
STKGPSVFPLAPSSKSTSGGTAALG**C**LVKDYFPEPVTVSWNSGALTSGVHTFPAVLQSSG
LYSLSSVVTVPSSSLGTQTYI**C**NVNHKPSNTKVDKKVEPKS**C**DKTHT**C**PP**C**PAPEL

>Cetuximab light chain
DILLTQSPVILSVSPGERVSFS**C**RASQSIGTNIHWYQQRTNGSPRLLIKYASESISGIPS
RFSGSGSGTDFTLSINSVESEDIADYY**C**QQNNNWPTTFGAGTKLELKRTVAAPSVFIFPP
SDEQLKSGTASVV**C**LLNNFYPREAKVQWKVDNALQSGNSQESVTEQDSKDSTYSLSSTLT
LSKADYEKHKVYA**C**EVTHQGLSSPVTKSFNRGE**C**

Figure S1. Protease cleavage sites within Cetuximab Fab' heavy and light chain amino acid sequences. Related to Figure 1C,D and Figure 2 B. Cleavage sites within Fab heavy chain (PDB 1yy9 and https://www.ebi.ac.uk/pdbe/entry/pdb/1yy9/protein/3) and light chain sequences were identified by using https://web.expasy.org/cgi-bin/peptide_cutter/peptidecutter.pl Potential Cathepsin K- cleavable AA sites are shown in yellow. Cathepsin B – cleavage site is shown in blue. Trypsin (model protease) -cleavage sites : lysine residues are shown in red (please note that K residues are also sites of acylation with the 800CW dye, arginine residues are shown in green.

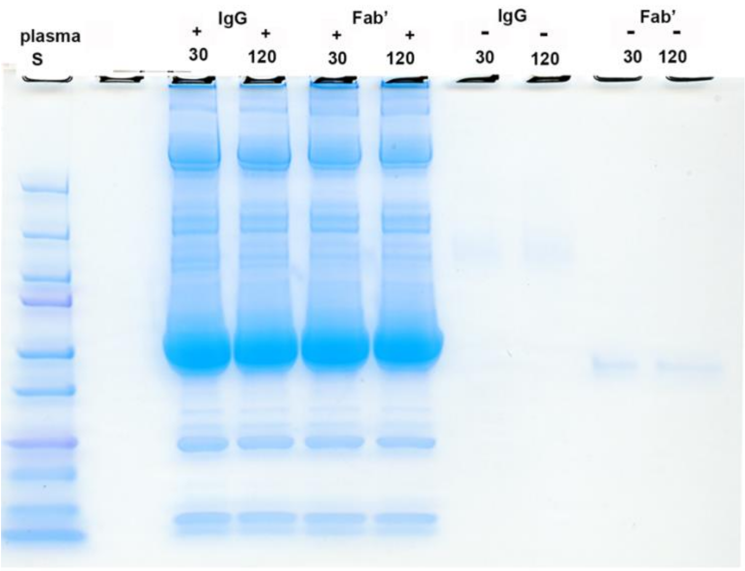

Figure S2. Coomassie G-250 (GelCode™ Blue Stain Reagent Thermo Scientific™) staining of the gel shown in Figure 2 to demonstrate the loading of the gel with samples containing mouse blood plasma vs. control ones that did not contain plasma. Related to Figure 2A. S- molecular weight standards, Lanes marked as IgG contained Cetuximab, Fab'- Cetuximab fragment, incubated either for 30 or 120 min in the presence (+) or the absence (-) of mouse plasma.

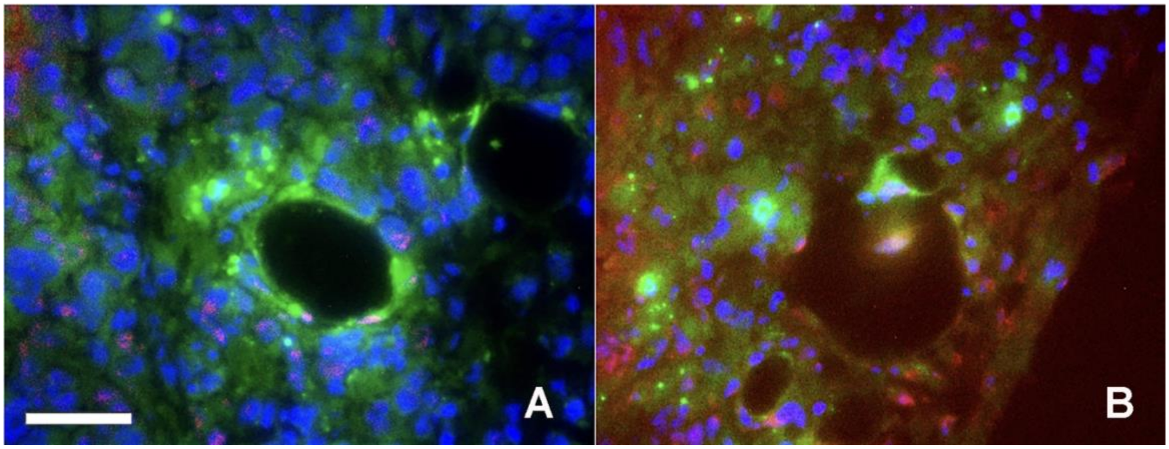

Figure S3. Fluorescent microscopy of tumor frozen sections. Tumor margin area staining with anti-mouse CD31 (green), nuclei – DAPI (blue), NIR fluorescence (800CW, red). IRDye 800CW fluorescence was excited using a 780 nm, 170 mW Collimated LED (ThorLabs). A- T2.1 CRISPRi-attenuated tumor, B – wild type MDA-MB-231 tumor. Scale = 20 µm. Related to Figure 8 (E and F).
